# Olfactory-cued navigation in shearwaters: linking movement patterns to mechanisms

**DOI:** 10.1038/s41598-018-29919-0

**Published:** 2018-08-02

**Authors:** Milo Abolaffio, Andy M. Reynolds, Jacopo G. Cecere, Vitor H. Paiva, Stefano Focardi

**Affiliations:** 1 Department of Physics, University of Padova, Padova, Italy; 2 ISC-CNR, via Madonna del Piano 10, Sesto Fiorentino, 50019 Italy; 3 Rothamsted Research, Harpenden, AL5 2JQ UK; 4 ISPRA, via Ca’ Fornacetta 9, 40064 Ozzano dell’Emilia, Italy; 5 LIPU, LIPU-Birdlife Italy, via Udine 3/a, Parma, 43122 Italy; 6 MARE, Marine and Environmental Sciences Centre, Department of Life Sciences, University of Coimbra, Coimbra, 3004-517 Portugal

## Abstract

After foraging in the open ocean pelagic birds can pinpoint their breeding colonies, located on remote islands in visually featureless seascapes. This remarkable ability to navigate over vast distances has been attributed to the birds being able to learn an olfactory map on the basis of wind-borne odors. Odor-cued navigation has been linked mechanistically to displacements with exponentially-truncated power-law distributions. Such distributions were previously identified in three species of Atlantic and Mediterranean shearwaters but crucially it has not been demonstrated that these distributions are wind-speed dependent, as expected if navigation was olfactory-cued. Here we show that the distributions are wind-speed dependent, in accordance with theoretical expectations. We thereby link movement patterns to underlying generative mechanisms. Our novel analysis is consistent with the results of more traditional, non-mathematical, invasive methods and thereby provides independent evidence for olfactory-cued navigation in wild birds. Our non-invasive diagnostic tool can be applied across taxa, potentially allowing for the assessment of its pervasiveness.

## Introduction

Tubenosed species are pelagic birds nesting in colonies located on islands and they are obliged to wander in visually featureless seascapes when foraging. Thus these species need efficient navigational systems to return quickly and reliably to their home, especially during chick-rearing. Bonadonna *et al*.^1^ reviewed the mechanisms of orientation used by pelagic birds and hypothesised that their remarkable navigational capabilities can be attributed to the birds learning an odor landscape. Benhamou *et al*.^2^ showed that white-chinned petrels, *Procellaria aequinoctialis*, were able to home after a displacement greater than 300 km using non-geomagnetic site-dependent information. Gagliardo *et al*.^3^ subsequently reported on olfactory navigation in Cory’s shearwaters (*Calonectris borealis*) in the Azores, and Reynolds *et al*.^4^ showed that the flight patterns of three species of shearewaters (*C. borealis*, *C. diomedea* and *C. edwardii*) in both the Atlantic Ocean and the Mediterranean Sea were consistent with a reliance on olfactory-cued navigation. Further Pollonara *et al*.^5^ showed that olfactory information is even used for homing in the Mediterranean, a sea with plenty of landmarks. Shearwaters are responsive to dimethylsulphide (DMS)^6^ which is a good candidate cue for olfactory navigation, as it is produced in large amounts by the phytoplankton. The importance of DMS in the foraging ecology of pelagic birds has been reviewed by Nevitt^7^. Indeed DMS characterises sea areas of high productivity and thus rich foraging grounds.

Anatomical evidence provides further support for the crucial role of olfactory cues for navigation in pelagic birds. Corfield^8^, for example, showed that the relative size of the olfactory bulb is especially large in *Procellariformes*, thus it is hardly surprising that olfactory orientation and navigation has been preferentially researched in marine birds. Beyond *Procellariformes*, the hallmarks of olfactory navigation has been also found in a few other species of wild birds such as swifts, *Apus apus*^9^, starlings, *Sturnus vulgaris*^10^, catbirds *Dumetella carolinensis*^11^ and lesser black-backed gull *Larus fuscus*^12^.

If it really exists, then how does olfactory navigation work? This very interesting question has been analysed only in the context of the navigation mechanisms of homing pigeons (*Columba livia*) (see^13,14^ and references therein). It has been hypothesised that these birds use ratios of concentration between dominant odors in the landscape, which appear more stable in time than odor concentration itself^15^. These authors showed that at least 16 chemical compounds were present in their study area in Germany, which resulted in at least 120 potentially-usable ratios. It is thought that homing birds associate odor bouquets with the direction of the prevalent winds. Once far from home, birds recognise the local bouquet and home accordingly.

This idea was used by Reynolds *et al*.^4^ who developed a model of odor cued navigation showing that the resulting flight patterns should be a special form of truncated Lévy walk. That is, the flight patterns are predicted to consist of sequences of near-unidirectional flight. The lengths *l*, of these near-unidirectional flights, are distributed according to a power-law with an unusual double-exponential truncation, that is a defining characteristic of the theory. Reynolds *et al*.^4^ examined the flight patterns of 210 shearwaters and found that 69% were consistent with this theoretical expectation for a reliance on olfactory-cued navigation.

Here we make more stringent tests of the theory developed by Reynolds *et al*.^4^, trying to detect the signature of olfactory navigation in the trajectories of shearwaters recorded using modern GPS technology. We go beyond the previous analyses of Reynolds *et al*.^4^ by showing that the bi-exponential truncated power-law, hereafter called BETPL, in flight length distribution:

1 $$p(l)=N{l}^{-\mu }{e}^{-{\lambda }_{1}l}{e}^{-{\lambda }_{2}/l}$$

is dependent on the local wind conditions, as would be expected if navigation were indeed odor-cued. More specifically, the fitted distributions should be characterized by a universal exponent, *μ*, whose value, 3/2, is independent of ecological context^4^. The exponential decay rates, *λ*_1_ and *λ*_2_, are instead related to atmospheric turbulence as shown in the next section. The quantity *N* is just a normalization factor. This is an example of “free Lévy flights hypothesis” as proposed by Reynolds^16^ which claims that Lévy flights arise spontaneously from interactions between animal behaviour and external conditions and are retained if they are advantageous. This approach goes beyond the classical Lévy flight foraging hypothesis^17^, a theory developed in order explain the flight patterns of wandering albatrosses which are thought to optimize the search for food^18,19^ (see Edwards *et al*.^20^ for a countrary view).

In order to test the olfactory map hypothesis we (i) evaluated how many trajectories are double exponentially truncated Lévy flights in a sample of shearwaters encompassing 7 populations of three species (*C. diomedea*, *C. borealis* and *C. edwardsii*) (ii) estimated the values of *μ*, *λ*_1_ and *λ*_2_ using likelihood methods (iii) investigated the correlations between wind speed and these three parameters and (iv) estimated correlations between *μ*, *λ*_1_ and *λ*_2_, and biological and environmental conditions.

To summarize the rationale: (1) there is a mathematical theory of odour-cued navigation that is rooted in turbulence theory and describes how odours disperse in the atmospheric boundary-layer; (2) this theory makes very a distinct and seemingly peculiar prediction, namely that flight-segments are distributed accordingly to a doubly exponentially-truncated power-law with power-law exponent 3/2 and where the exponential truncation parameters are wind-speed dependent; (3) The aim of this paper is to test these hypotheses. If verified, we have added a new evidence to the growing body of evidence^3,5,21^ which suggests that shearwaters do indeed rely on olfactory-cued navigation. Thus our approach complements more traditional studies with the application of a new method of data analysis.

Further we show that the hypotheses are indeed consistent with empirical data and thereby provide a strong evidence for cognitive odor map^22^ navigation in wild birds, which interestingly is independent from the previous evidences derived by manipulative experiments.

## The Model of Olfactory-Cued Navigation

**Figure 1:**
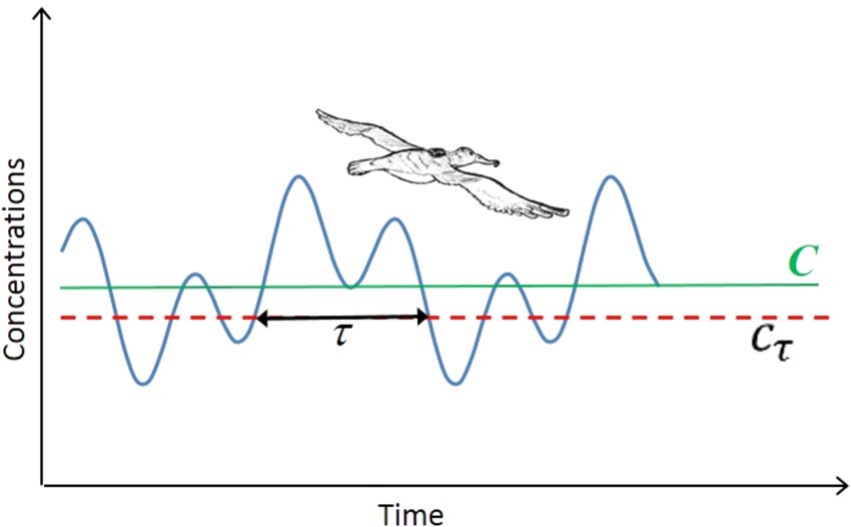
A sketch of our model assumptions, a GPS-tagged bird is flying and the odor concentration (grey continuous line) *c*_*τ*_ can be above or below the detection threshold (red dotted line). During the time *τ* the bird is in contact with the map and may move towards its target. The green line denote the mean odor concentration *C*. Note that the bird is expected to lose contact with the odour information because of atmospheric turbulence.

Air flows are inherently unstable and as a consequence, smooth, regular movement soon become large eddy (swirling or vortex) movements. These large eddies subsequently break-up into smaller eddies which are in turn unstable. This leads to a cascade of energy from the largest eddies to the very smallest eddies, where energy is eventually dissipated as heat. In between the largest and smallest eddies, the replication of eddies is the same at every spatial scale, and this results in “self-similar” flow patterns (i.e. swirls within swirls). Mathematically such patterns are characterized by power-laws^23^. But this characterization can only apply to intermediate eddies and must be truncated at the largest and at the smallest eddies. These eddies also transport and break-up odour packets. Odour concentrations like eddy sizes are therefore expected to have doubly-truncated power-law distributions just as predicted by Reynolds *et al*.^4^. This must have a significant influence on olfactory-cued navigation because odour concentrations are only ever present intermittently. Reynolds *et al*.^4^ suggested that when the odors are continually present above the threshold of detection, *C*_*τ*_, then the ‘odor map’ is available and the birds can be expected to maintain nearly unidirectional flight in the presumed target direction. But when the odor concentration falls below this level we assumed that the birds are without their map and so effectively lost. They may change course either because they become disoriented or because they are attempting to re-establish contact with the odor map (Fig. 1). In conclusion, Reynolds *et al*.^4^ showed that turbulence theory predicts that the lengths of the unidirectional flights will be distributed accordingly to exponentially-truncated power-laws of the form given in equation 1. This prediction is not model specific, as it arises generally in physical realistic models of turbulent dispersal of odors (See Supplementary Section S1). Celani *et al*.^24^ deduced a similar model starting with a different set of assumptions. The model is characterized by 3 parameters: *μ*, the Lévy exponent, *λ*_1_ and *λ*_2_. 1/*λ*_1_ is the scale of truncation on very long steps while *λ*_2_ is the scale of truncation on the small step lengths. According to Reynolds *et al*.^4^.

2 $${\lambda }_{1}\propto 1/T$$

and

3 $${\lambda }_{2}\propto T\frac{{c}_{\tau }^{2}}{{C}^{2}},$$

where *T* is the autocorrelation timescale, i.e., the typical durations over which concentrations remain significantly correlated, *c*_*τ*_, is the threshold concentration above which birds may detect the odor, and *C* is the mean odor concentration over time. We expect both *T* and *C* to be dependent on atmospheric conditions, especially on the mean wind speed. The existence of such dependence can be seen with the aid of a simple heuristic argument. Imagine, for example, a patch of ocean with area *L*^2^ releasing odor in to atmosphere at a rate *F* which subsequently becomes dispersed by turbulence up to a height *H* in the atmospheric boundary-layer. The total quantity of odor released from the patch during a time interval of duration *t* is *Q* = *FL*^2^*t* and this will become distributed throughout a volume of air *V* = *LHUt*, where *U* is the wind speed. The mean odor concentration is then $C=Q/V\propto F/U$. According to existing literature^25^, empirical observations suggest that the flux of DMS and so presumably other volatiles is itself dependent upon the mean wind speed, such that *F*∝*U*^*γ*^ where estimates for the characteristic exponent, *γ*, range between about 2 and 3. The autocorrelation timescale will also depend on the mean wind speed *U*. Hence *λ*_1_ and *λ*_2_ must also depend on the mean wind speed because they depend on the autocorrelation time scale and the mean odor concentration. We look for these relationship by regressing our estimates for *λ*_1_ and *λ*_2_ with wind speed data, as described in the next section. A biological interpretation of the model is reported in Supplementary Section S2.

## Methods

### Study areas and field methods

Scopoli’s shearwater *Calonectris diomedea* (Cd), Cory’s shearwater *C. borealis* (Cb) and Cape Verde shearwater *C. edwardsii* (Ce) are three closely related *Procellaridae* species breeding in the Mediterranean, North Atlantic Ocean, and Central Atlantic Ocean, respectively. These burrowing seabird species generally feed on pelagic fish, cephalopods and crustaceans^26^, but also fishery discards^27^. Birds were tagged using different data-loggers, all recording GPS-positions every 10 minutes. For more details about tag types, their deployment and birds’ trajectories see Reynolds *et al*.^4^. The birds’ trajectories are the same analyzed in Reynolds *et al*.^4^. Fieldwork on Cd was carried out during both the incubation and chick-rearing periods at three Mediterranean colonies: Linosa island ($35^\circ 52^{\prime} {\rm{N}}$; $12^\circ 52^{\prime} {\rm{{\rm E}}}$), the Tremiti Archipelago ($42^\circ \,08^{\prime} {\rm{N}}$; $15^\circ \,31^{\prime} {\rm{E}}$), and La Maddalena Archipelago ($41^\circ 13^{\prime} {\rm{N}}$; $9^\circ 24^{\prime} {\rm{E}}$) (Fig. S2 in Supplementary Section S3). Fieldwork on Cb was carried out during chick-rearing period at three north Atlantic islands: Corvo ($39^\circ 40^{\prime} {\rm{N}}$; $31^\circ 06^{\prime} {\rm{W}}$), Berlenga ($39^\circ 24^{\prime} {\rm{N}}$; $9^\circ 30^{\prime} {\rm{W}}$) and Selvagem Grande ($30^\circ 08^{\prime} {\rm{N}}$; $15^\circ 51^{\prime} {\rm{W}}$). Finally, fieldwork on Ce was carried out during both incubation and chick-rearing periods at Raso islet ($16^\circ 36^{\prime} {\rm{N}}$; $24^\circ 35^{\prime} {\rm{W}}$). Data have been uploaded to The Seabird Tracking Database managed by BirdLife International (http://www.seabird-tracking.org/).

### Wind data

The wind data were downloaded from the NOAA^28^ web site from the *rerdapp*^29^ package for R^30^. The data comes from satellite measurements, have spatial resolution of 0.5 degrees and time resolution of 6 hours and are indicative of the wind speed at 10 meters above the sea level. We perform for each wind direction a simple linear interpolation in space and time in order to estimate the wind speed at the spatio-temporal coordinates of the birds (other methods like inverse distance weighted or spline were tested on a subset and lead to not relevant differences). For completeness we make regression using not only the mean wind speed <*v*>, but also with other weighted averages like $\sqrt{ < {v}^{2} > }$ or $\sqrt[3]{ < {v}^{3} > }$, in order to test the importance of the fluctuations and in particular the stronger gusts of the wind. The most significative will turn out to be the arithmetic mean and the others will not be presented in later analyses.

### Identification of turning points

To extract the step length distributions from the birds’s trajectories, the research community is now widely using the methods of Humphries *et al*.^19^. This method is used because turning points can be identified in an unambiguous way without resorting to arbitrarily defined thresholds for turning angles because  it preserves power law statistics. Each trajectory is projected along the two coordinate axes to create two one dimensional artificial trajectories. Turning points in 1D trajectories are straigthforwardly defined as changes in the direction of travel. The statistics of step lengths are then grouped together. Since our expectation for the birds step distribution is not a pure power law, before starting the analysis we performed a validation of the selected method^19^ with synthetic data that are reported in supplementary materials (see Supplementary Section S4). We also show analytically that the method works for exponentially truncated power laws (see Supplementary Section S5).

### Distribution fitting

In power law analysis it is necessary to fix a lower bound on flight lengths^31^, lim_*a*_, which in our case has the advantage of removing small scale flights behaviour that are not related with navigation. The maximum likelihood is estimated for the model parameter *λ*_1_, *λ*_2_ and *μ* in the domain ${\rm{\Theta }}=(0,{\lambda }_{1}^{M})\times (0,{\lambda }_{2}^{M})\times ({\mu }^{m},{\mu }^{M})$, with ${\lambda }_{1}^{M}=0.2$, ${\lambda }_{2}^{M}=3$, *μ*^*m*^ = 1.0 and *μ*^*M*^ = 3.0, with M,m denoting the maximum and minimal values used to define the grid of parameter variation that is sampled with Δ*λ*_1_ = 0.002, Δ*λ*_2_ = 0.005 and Δ*μ* = 0.01. *A posteriori* we verified that the ${\lambda }_{1}^{M}$ and ${\lambda }_{2}^{M}$ exceed our estimates in the majority of cases.

The best lim_*a*_ for every bird travel is unknown *a priori* and should be checked with a goodness of fit test^19^. We tested a wide range of lim_*a*_ values, namely (0.05, 0.10, 0.20, 0.30, 0.40, 0.50, 0.60, 0.70, 0.80, 0.90, 1.00, 1.20, 1.40, 1.60, 1.80, 2.00, 2.50, 3.00 km) where the extreme values are never attained in our sample.

For the sake of realism we assumed that actual birds may have a preferred direction of movement, for instance toward the colony or the foraging grounds. To remove any bias due to preferred directions on the estimated statistics, we replicated the analysis after a rotation of the fixes for 4 different angles *θ*: 0, 15, 30, 45 degrees around the initial point and we checked the consistency of the fitted statistics. The maximum step length allowed is set to 600 Km, the maximum value observed in our dataset. For every trajectory we tested 72 different parametrization: the 18 values of lim_*a*_ and 4 values of *θ*. We use a modified Kolmogorov-Smirnov gof test to select the best one among these parametrizations. More specifically we used a version of the Kolmogorov-Smirnov test that ensures an uniform sensitivity across all the distribution range^31^. We verified that this approach is appropriate using synthetic trajectories, see Supplementary Section S4. In order to verify the robustness of our method we perfomed a sensitivity analysis and we showed that the parameters *β*_1_ and *β*_2_, the regression coefficients between *λ*_1_, *λ*_2_ in function of the mean wind are estimated very consistently, (Supplementary Section S6).

The likelihood distribution for the parameters *λ*_1_, *λ*_2_ and *μ* is often highly skewed and the proper estimator should be the mean likelihood instead of the maximum likelihood^32^. Confidence intervals for the three parameters were computed using the 2.5% and the 97.5% percentiles. Once verified that *μ* is close to 3/2 all the analysis were repeated keeping *μ* fixed at this reference value. The reason being that the measure of *μ*, *λ*_1_ and *λ*_2_ are highly correlated and fixing *μ* is useful to reduce bias and increase precision of *λ*_1_ and *λ*_2_ as well to improve the statical power of our test (as seen in Supplementary Section S4 for synthetic trajectories). The maximum likelihood in this new parametrization is evaluated in the domain ${\rm{\Theta }}=(0,{\lambda }_{1}^{M})\times (\mathrm{0,}\,{\lambda }_{2}^{M})$, with ${\lambda }_{1}^{M}=0.2$, ${\lambda }_{2}^{M}=3$, sampled with Δ*λ*_1_ = 0.0005 and Δ*λ*_2_ = 0.0005 so sampling the *λ*_1_, *λ*_2_ with an order of magnitude more in accuracy than the variable *μ* cases.

### Comparing with alternative models

We compare our model with competing models such a bi-exponential, simple power laws, power law with only high scale truncation, simple exponential and an other distribution obtained by the sum of a BETPL and an exponential. These alternative models have been clearly observed across taxa and are biological meaningful: a good fit to a bi-exponential would indicate a bimodal search; a good fit to a power law would indicate a Lévy search pattern; a exponential would be indicative of a single-scale search. For every model we optimize the goodness-of-fit with respect to lim_*a*_ and *θ*. Then we determine which model is more appropriate for our data.

### Hypothesis testing

We used a standard linear regression analysis on a log-log plot to test our hypothesis (*lm* function in package *stat* for^30^). We also compute the prediction and the confidence interval of the regression coefficient that we use to put in evidence the presence of eventual outliers in the sample. The result may depend on the duration of the excursion considered in the analysis. In the main text we report one specific subsetting of our data, but a more general analysis relative to all possible subsettings, from 2 to 6 days, is reported in supplementary materials, Supplementary Section S6.

### Biological covariates

The biological and ecological covariates (sex: male or female, period: incubation, chick rearing, Marine region: Atlantic Ocean or Mediterranean and colony identity) are sequentially added to the reference models: $\mathrm{log}({\lambda }_{1})\sim \,\mathrm{log}(\bar{v})$ and $\mathrm{log}({\lambda }_{2})\sim \,\mathrm{log}(\bar{v})$ provided the esplicatory variables are reasonably independent. We report models which exhibit a ${\rm{\Delta }}\mathrm{AIC}\le 4$.

## Results

### Model discrimination

A preliminary analysis have showed that the bi-exponential models fit our data much better than any other model alternatives to the BETPL. Thus in Table S2 and S3 in Supplementary Section S7 we report only the comparison between the bi-exponential and the BETPL models. The BETPL were better supported than were the bi-exponential in the the 89% of cases if we fit all three parameters of BETPL and in the 81% of cases if we fit the BETPL fixing *μ* = 3/2. In all following analysis we use only the trajectories which fit the BETPL model.

### The power law exponent *μ*

**Figure 2:**
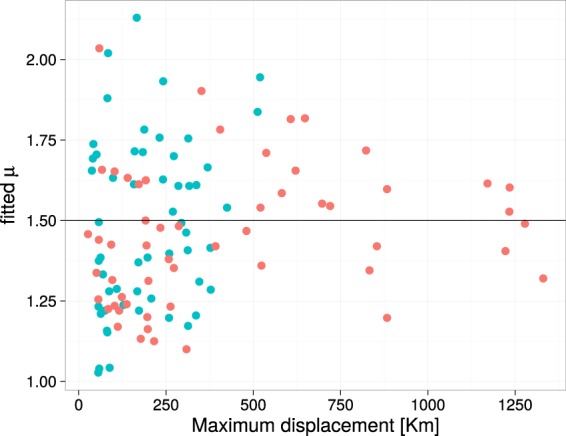
Fitted value of *μ* plotted against the maximum displacement reached from the colony. The horizontal line is the predicted value for *μ*. Data relative to seven colonies in the Atlantic ocean (Red dots) and in the Mediterranean sea (blue dots).

The first prediction of the model of Reynolds *et al*.^4^ is that *μ*, the exponent of the power law in BETPL, is 3/2 and is independent of the wind. In Fig. 2 we plot the fitted *μ* of every trip against the maximum displacement from the colony. The estimated *μ* value converges to the predicted value 3/2 as the displacement increases (see Fig. 2). A similar result can be obtained using the duration of the trip as independent variable, as we known that trip duration is correlated with the maximum distance from the colony^33^. These convergences are due to the increase of statistical power as can be seen in Fig. S17 in Supplementary Section S8 where is plotted the mean squared error from the predicted value 1.5 for different subsetting in the maximum distance reached from the colony. As predicted correlation between the mean wind and the fitted *μ* are very low (*r* = 0.12, *P* = 0.23, df = 108) and no pattern is evident by visual inspection (see Supplementary Section S7). This is true even after subsetting the datasets for trajectories that last more than 2, 3, 4, 5 or 6 days.

### Large scale truncation: *λ*_1_

**Figure 3:**
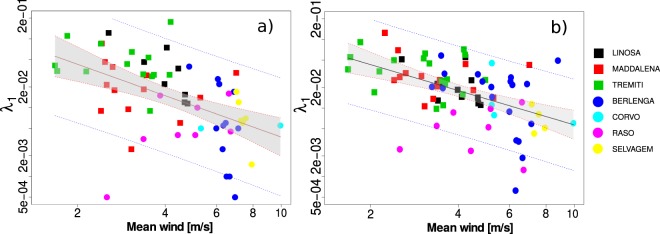
Data relative to seven colonies in the Atlantic ocean (dots) and in the Mediterranean sea (squares). (**a**) Log-log plot of the mean wind against *λ*_1_ for trajectories that last more than 4 days. (**b**) Log-log plot of the mean wind speed against *λ*_1_ fitted with *μ* = 3/2 for trajectories that last more than 2 days. The blue line is the 95% confidence limits for the mean predicted values and the red line is the 95% confidence limits of the individual predicted values.

Figure 3a shows a log-log plot of the fitted *λ*_1_ over the mean wind speed for trips that last more than 4 days. The fitted regression coefficient is negative and highly significant (*β*_1_ = 1.5 ± 0.3, *P* < 0.0001). This result is not specific for the subsetting used, as different subsettings yield similar results as detailed in Fig. S14. Similar results were also obtained after fixing *μ* = 3/2 (Fig. 3b). The fitted *β*_1_ is −1.26 ± 0.26 (*P* < 0.0001). As expected by the validation in Supplementary Section S4, the results changes only slightly respect of the ones plotted when *μ* is estimated from the data (Fig. 3a). As expected the regression is negative, small *λ*_1_ values are typical of area of great turbulence.

### The short scale truncation *λ*_2_

**Figure 4:**
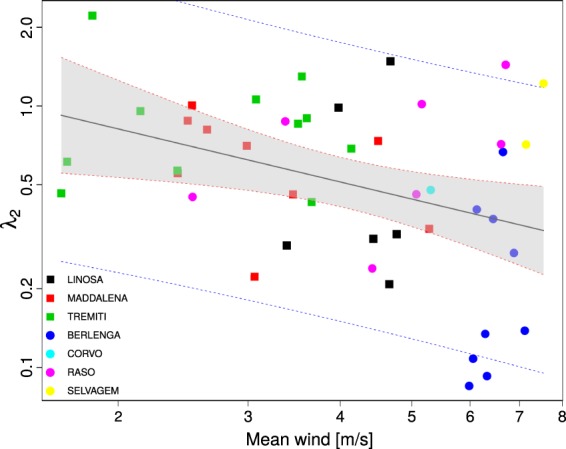
Data relative to seven colonies in the Atlantic ocean (dots) and in the Mediterranean sea (squares). Plot of the mean wind against *λ*_2_ for trajectories that last more than 4 days.

If we estimate *λ*_1_, *λ*_2_ and *μ* together then we obtain a very poor fit of *λ*_2_ as a function of the mean wind speed: (*β*_2_ = −0.2 ± 0.3, *P* = 0.55, *df* = 49). Instead if we fix *μ* = 3/2 then our statistics improve and a negative relationship between *λ*_2_ and the mean wind speed becomes evident as can be seen in Fig. 4. Although very noisy the fit indicates a clear negative *β*_2_ (−0.7 ± 0.3, *P* = 0.012, df = 43). Figure 4 has been computed for trajectories longer than 4 days but similar patterns can be obtained using different subsettings (see Fig. S16): the relationship remains negative and significant but the estimates of *β*_2_ are not as stable as the estimates for *β*_1_. It is clear that outliers are concentrated at high wind speeds. Indeed these are birds from the Atlantic Ocean. In particular the lowest *λ*_2_ values were observed at Berlenga.

### Biological covariates

**Figure 5:**
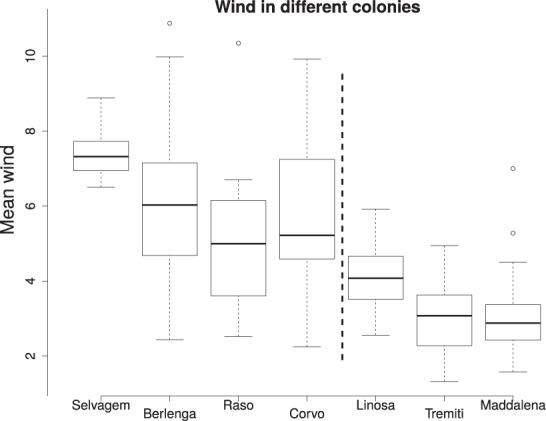
Boxplot of the wind speed recorded during bird excursions on 7 different colonies. The vertical line separates the Atlantic (left) from the Mediterranean (right) colonies.

In Fig. 5 we show that the mean wind speed is dependent on location, being much larger in the Atlantic ocean than in the Mediterranean sea (*t*_108_ = 11.4, *P* < 0.0001). Because of this correlation we test only models with period and sex, Table 1.

**Table 1:** Difference of AIC between the basic model and the other tested model for a subsetting of days that last more than 4 days.

Variable	Model	Δ AIC
*λ*_1_	log(<*v*>) + log(<*v*>):sex	1.345
*λ*_1_	log(<*v*>) + log(<*v*>):period	1.652
*λ*_1_	log(<*v*>) + log(<*v*>):sex + sex	3.105
*λ*_1_	log(<*v*>) + log(<*v*>):period + period	3.522
*λ*_2_	log(<*v*>) + log(<*v*>):period	0.563
*λ*_2_	log(<*v*>) + log(<*v*>):sex	2.000
*λ*_2_	log(<*v*>) + log(<*v*>):period + period	2.545
*λ*_2_	log(<*v*>) + log(<*v*>):sex + sex	3.944

The biological covariates have no significant effect on both coefficients *λ*_1_ and *λ*_2_, see Table 1. The best alternative model for *λ*_1_ includes the effect of sex that turns out to be not significant (*t* = −1.0 *P* = 0.3). The best alternative model for *λ*_2_ includes the effect of period that turns out to be not significant (*t* = 1.1 *P* = 0.3). For *μ* no model produces a significant *p*-value, indicating a complete independence of *μ* on all other predictors (data not presented).

### Possible mechanistic wind effect

We have tested the eventual presence of direct effect of wind speed on truncation parameters independently of odour cued navigation in Supplementary Section S9. We found a scarce, albeit significant, effect of wind speed of both head- and tail- winds on the value of *λ*_1_ while *λ*_2_ appears unaffected by head- and tail- winds.

## Discussion

With the present study we show that at-sea flight patterns of foraging shearwaters are clearly wind-dependent as suggested by the proposed olfactory navigation mechanism. Our methods of analysis are novel and inspired by a mathematical theory for how odours disperse within the atmospheric boundary-layer and accounts for the complex ways in which turbulence distorts and disperses packets of odour. Despite its mathematical complexity, the theory is useful to biologists and ecologists interested in odour-cued navigation because it makes definitive predictions that can be tested by careful analysis of bird telemetry, using statistical methods that are now standard in the literature on Lévy walks^19,31^. The hypothesis that shearwaters rely on odour-cues for navigation then becomes a sharply defined, falsifiable statement about the distribution of flight-segments. Our model distribution contains three parameters. One is uniquely determined by the theory, and is pure a number, 3/2, the other two, *λ*_1_ and *λ*_2_ are predicted to depend on the mean wind speed. It is thus unlikely that good fits to our model predictions can be attributed to other processes. We find that the characteristic power law exponent *μ* is clearly distributed around the expected value of 3/2 and is independent of the mean wind speed, as expected, while the other two parameters *λ*_1_ and *λ*_2_ depends on the wind. It is worth nothing that these dependences are not generic ones but are specific to our theory in sign and size. Our results provide clear and compelling evidences of olfactory-cued navigation in shearwaters and bolsters significantly the previous findings^4^ who did not test for wind-speed dependences and complements the evidence, obtained using more traditional methods^3,5,21^. We thereby linked flight patterns to underlying navigation mechanism for the first time.

According to the mechanism initially proposed by Kramer and Gustav^34^ the navigation should be a very precise and predictable tool: once the animal is able to get its coordinates, it can use a compass to attain its target; on the contrary the mechanism we proposed is strongly stochastic since it is influenced by the often unpredictable turbulence of the atmosphere. On one hand turbulence is indispensable for odor navigation because it carries information to the birds. On the other hand too much turbulence might reduce the navigation ability due to the high variability of the odor signal. This stochasticity might explain, at least in part, the variability which has been observed in experiments with homing pigeons. Indeed Walraff *et al*.[13, Chapter~3.4] concluded that “The angular dispersion of bearings appeared to reflect stochastic noise and is most reasonably compatible with the hypothesis that no one pigeon was able to gain clear-cut information on the direction of home, because noise was inherent already in the environmental signals providing positional information.” Note that our model is not a model for chemotaxis, i.e. the movement along stable gradients in odour concentration. In the atmospheric boundary-layers there are no such gradients since odours are being continually distorted into filaments and puffs, and dispersed by turbulence. The odours form a mosaic that is continuously fluctuating in both time and space. For this reason a bird performing only long-range chemotaxis would not obtain any information on his position with respect to the target and would not be able to navigate.

The observed dependencies of *λ*_1_ and *λ*_2_ are crucial for detecting olfactory cued navigation because a *μ* value of 3/2 can be also determined by other mechanisms. Further evidence that our interpretation is correct is the exclusion from most of the studied trajectories of alternative movement patterns such as simple exponential, bi-exponential, power-law and single truncated power law. To our knowledge there is no other mechanism which could explain the observed relationship of *λ*_1_ and *λ*_2_ with wind speed. The estimation of *λ*_2_ differently to the estimation of *λ*_1_ suffers from a lack of resolution in our trajectories. Indeed a sampling of 10 minutes are a compromise between resolution and battery duration. We expect that newest gps-logger generation may allow for a denser sampling of trajectories which may improve the estimation of *λ*_2_. Small scale behavior unrelated to navigation^35^ is one such source of noise in the estimated *λ*_2_. Furthermore our simulations have showed that *λ*_2_ is the most difficult parameter to be estimated. Comparable results were obtained for both *λ*_1_ and *λ*_2_ where tested with different sub-settings, bootstrap and different methods of estimation (see Supplementary Section S6), all leading to comparable results. Three issues are worth noting. First, the trajectories collected in the Atlantic Ocean are mainly characterized by stronger wind speeds than Mediterranean trajectories. Second, few trajectories mainly from Raso and Berlenga behave as outliers of this relationship (however the presence of some outliers is expected from the analysis of synthetic trajectories, Supplementary Section S4). Third, we provided clear evidence (cf. Supplementary Section S6) that our results remain consistent changing the specific data selection criteria or methods of estimation. The mechanistic effect of wind on the flight pattern of shearwaters is not at all unexpected^36^ but its effect is much less relevant than the one determined by olfactory cue navigation.

According to Komolkin *et al*.^37^ a bird could use a hierarchy of orientation systems such as geomagnetic navigation at very long distances, olfactory at intermediate scales and piloting for short range movements. Surprising both Pollonara *et al*.^5^ and our study found olfactory navigation in birds from small Tyrrhenian islands, where we could have assumed that piloting can be quite effective. In our study we confirmed that shearwaters can rely on olfactory maps over distances of several hundreds of kilometers. It would be interesting to analyze the flight patterns of albatrosses that are wandering for thousand of kilometers in the southern oceans and determine whether olfactory navigation is the dominating mechanism also at such scales. Wandering albatrosses, *Diomedea exulans*, have been the forefront of the Lévy flight research^38^. This research led to an explosion of interest in Lévy flights as model of movement patterns of animals, because it was later reported that Lévy flights are optimal for probabilistic foragers^18^. Successive research^20^ have cast a shadow on Lévy flight research, and especially on wandering albatross behavior. However it was finally shown^19^ that the wandering albatross does in fact have flight patterns that Paul Lévy, after whom Lévy flights are named, would have appreciated.

The skeptical biologist may be reluctant to use a mathematical model, albeit sophisticated to demonstrate a mechanism of navigation and he would rely preferentially on manipulative experiments^3,5,21^. If our model is able to catch at least the essence of the navigation mechanism the result we obtained should be qualitatively consistent with those obtained by manipulative experiments. It is beyond the aims of this paper to review in depth the manipulative experiments performed on shearwaters but we tested our model against data from an independent experiment^5^. We showed (Supplementary Section S10) that the flight patterns of control birds, unlike those of anosmic birds, are consistent with theoretical expectation for olfactory-cued navigation.

Beside the importance of olfactory cued navigation for homing, demonstrated in *Procellariformies* and homing pigeons, recent researches have suggested that lesser black-capped gulls may use olfactory cued navigation during migration^12^. Our model may represent an appropriate tool to investigate this hypothesis on a large number of wild birds, especially when conservation concerns do not allow for experimental manipulation of large number of birds. More generally our model may be applied for other taxa such as ants^39^, seals and marine turtles where there is evidence for olfactory cued navigation at different spatial scales^40^.

## Electronic supplementary material

Supplementary methods
