# Supplementary material for: Olfactory-cued navigation in shearwaters: linking movement patterns to mechanisms
Source: Sci Rep. 2018 Aug 2;8:11590. doi: 10.1038/s41598-018-29919-0 (PMC6072774; doi:10.1038/s41598-018-29919-0)
Supplement: Supplementary file 1 — Supplementary methods [file 41598_2018_29919_MOESM1_ESM.pdf]

Olfactory-cued navigation in shearwaters: linking movement patterns to mechanisms: Supplementary materials

Milo Abolaffio[1,2], Andy M. Reynolds[3], Jacopo G. Cecere[4], Vitor H. Paiva[5], and Stefano Focardi[2]

[1]Department of Physics, University of Padova, Padova, Italy
[2]ISC-CNR, Sesto Fiorentino 50019, Italy
[3]Rothamsted Research, Harpenden AL5 2JQ, UK
[4]ISPRA, Ozzano dell'Emilia 40064, Italy
[5]MARE, Marine and Environmental Sciences Centre, Department of Life Sciences, University of Coimbra, Coimbra 3004-517, Portugal

July 26, 2018

S1 Power-laws with double-exponential truncation are a robust defining characteristic of olfactory-cued navigation

Reynolds *et al.* (2015) obtained an analytic expression of the step-length distribution by assuming that odor concentrations were exponentially distributed. The assumption is broadly consistent with observations of odor concentrations and our predictions provide good fits to the shearwater flight pattern data. Their key prediction is the occurrence of doubly-exponentially truncated 3/2 power-laws which Reynolds *et al.* (2015) took to be the hallmark of olfactory-cued navigation Here we provide evidence that this defining characteristic is not specific to the model of Reynolds *et al.* (2015) but instead arises generally from physically realistic models of olfactory-cued navigation.

To do this we have calculated the step-length under the assumption that odour concentrations are Gaussian distributed rather than exponentially distributed. This assumption is not very realistic but that does not matter because the goal here is to test the robustness of doubly-exponentially step-length distributions with respect to modelling assumptions. The calculation is long and tedious but mirror closely that of Nyberg *et al.* (2016). The end result can be expressed most succinctly as

$$C = Rank.Freq.Distr. = erf\left(\frac{c_0 e^{-t/T}}{\sqrt{2(1-e^{-2t/T})}\sigma_c}\right) \tag{1}$$

where for simplicity the detection threshold $c_T = 0$. It follows that the step-length (step-duration) distribution, $p = \frac{-\partial C}{\partial t}$, is given by

$$p(t) = \frac{c_0}{\pi \sigma_c} \frac{T^{1/2}}{t^{3/2}} e^{-c_0^2 T/(8\sigma_c^2 t)} \tag{2}$$

for long times. And so once again we have doubly-exponentially truncated 3/2 power-laws. This suggests that double-exponentially truncated 3/2 power-laws are a robust prediction that arises independently of modelling assumptions about how odours are distributed.

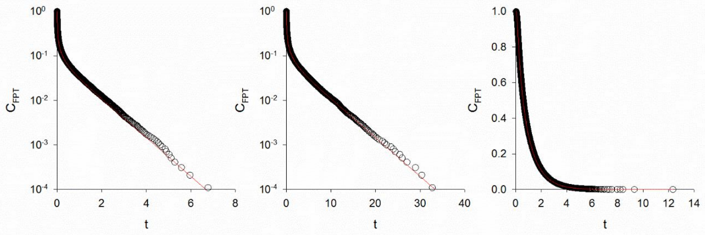

Figure S1: Figure Comparison of simulation data (o) and theoretical predictions (red lines) for a variety of parameter values showing that the theory works.

As in Reynolds *et al.* (2015) $\lambda_1 \propto 1/T$,and $\lambda_2 \propto T c_0^2$.

S2 Biological interpretation of double exponentially truncated power law function.

Figure S2 displays graphically the behaviour of the model predicted by our theoretical framework. The bold black curve represents the probability (ordinates) of performing a displacement whose lenght is reported on the abscissae. The parameters that identify our model are the exponent of the power law = -3/2, and the values of the exponential cut-offs (λ_1 and λ_2). Between the exponential cut-offs the curve is almost straight as it can be seen comparing it to the reference line of exponent -3/2. Note that a power law with exponent -3/2 is a Levy walk. If μ is larger the birds would change direction more often until to perform a brownian random walk when $\mu = 3$. On the other side, if μ is close to 1, the bird would move ballistically. Our model of olfactory navigation predicts, under very large set of conditions, that μ should be -3/2.

The cut off at small scale is λ_2 while the cut off at large scale is $1/\lambda_1$. The mechanism of odour navigation implies also an effect of the bird sensibility of odours and the average concentration of odours in the environment. All these factors are included in the definition of λ_2. Turbulence determines the odour concentration in the atmosphere. This parameter enter both in the definition of λ_2 and λ_1.

Note that the wind speed is proportional to $1/\lambda_1$ wich turn to say that the right cut of is proportional to the wind speed. These implies that the correlation coefficient between the logarithm of λ_1 and the logarithm of the wind speed should be -1. The prediction for λ_2 are less sharp: we predict a directed relationship with the wind speed but we cannot predict the value of the correlation coefficient, because we cannot know the values of the mean odour concentration that enter in the definition of λ_2.

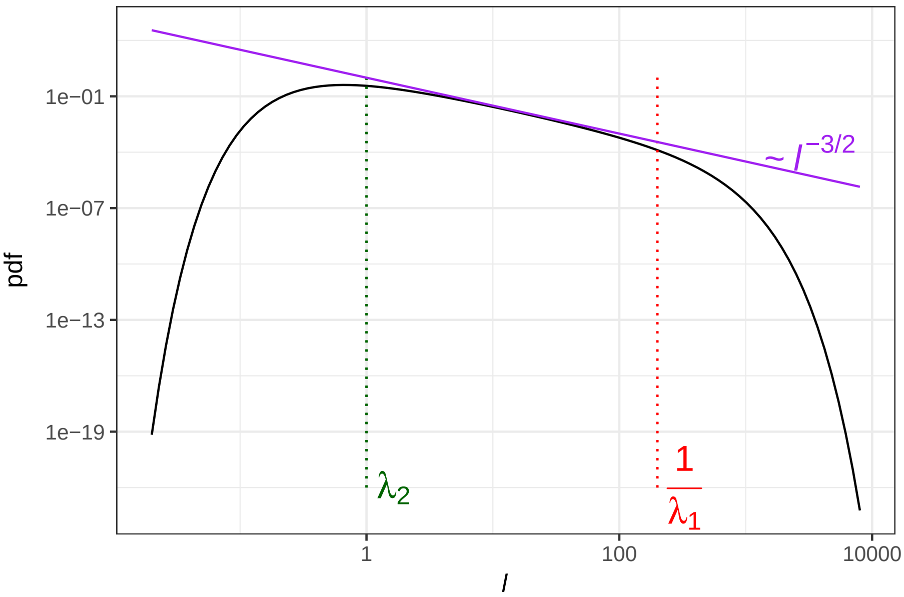

Figure S2: Log-Log plot of a double exponentially truncated power law eg the equation 1 in main text, with parameter $\lambda_2 = 1.0$ and $\lambda_1 = 0.005$. The continuous straight line is a power law with exponent -3/2.

S3 Study areas

In figure S3 we plot some trajectories of birds from all the different colonies considered in this study.

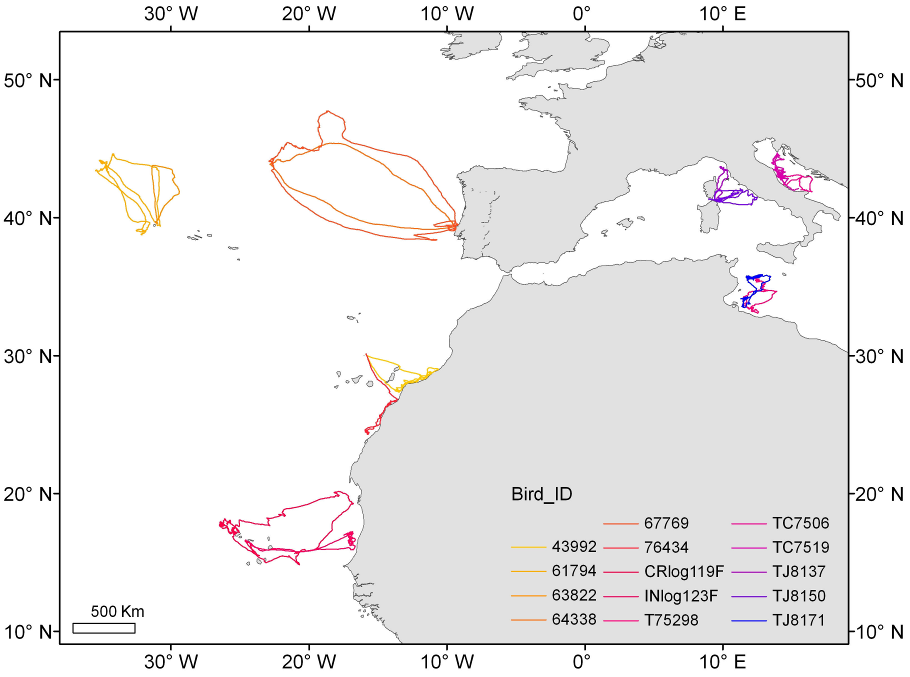

Figure S3: Some trajectories from the different colonies. The labels are a code identificative of the bird labels used to uniquely identify the birds.The figure was generate with ESRI ArcMap 10.1. available at http://support.esri.com/download/1913

S4 Validation of the method used by Humphries *et al.* (2013)

Although the method is presented as free of parameter choices, there are two parameters that indeed are hidden in the model. The first is $\lim_a$, the value of truncation of the projected data on the low scales and the second is θ the initial orientation of the reference systems. $\lim_a$ is needed in every data-set that is not a pure power law, since this method introduce distortions in the part of the pdf that is not power law distributed. Since our hypothesized distribution is not a pure power law on lower scale, we expect this parameter to be important. θ is instead important in the case of a preferential direction of travel. In order to estimate the best $\lim_a$ we perform 4 different tests: the Kolmogorov-Smirnoff test (KS), a version of the KS test that account for the tails[1] (KS_tail), the Von Mises test (VM) and the Anderson Darling test.

[1]The empirical distribution function F_n for n iid observations X_i is defined as $F_n(x) = \frac{1}{n}\sum_{i=1}^{n} I_{-\infty,x}(X_i)$ where $I_{-\infty,x}(X_i)$ is the indicator function, equal to $X_i \leq x$ and equal to 0 otherwise. A version of the K-S measure uniformly sensitive across the range for a given cumulative distribution function $F(x)$ is: $D_n = \max_{x>\lim_a} |F_n(x) - F(x)|/\sqrt{F(x)(1-F(x))}$

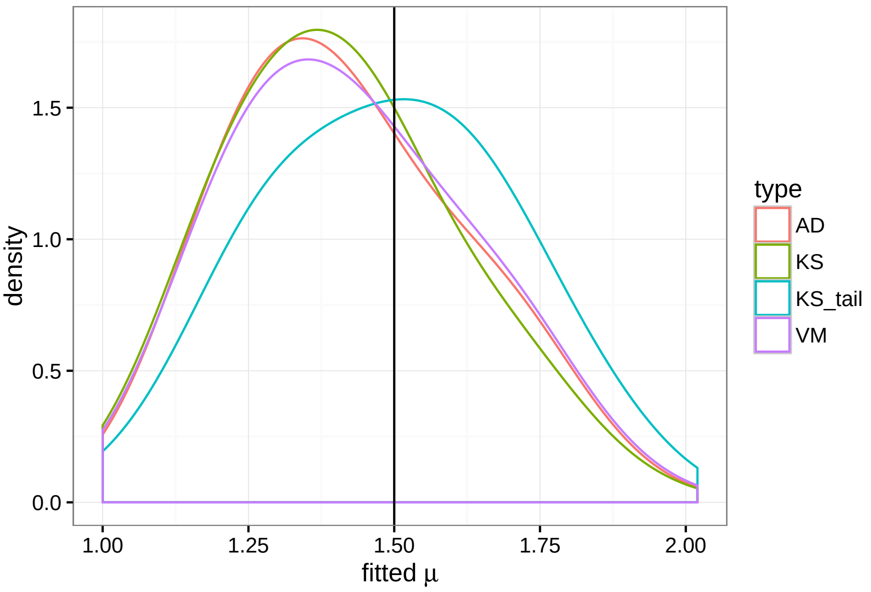

Figure S4: Distribution fitted μ obtained with the different gof methods. KS_tail result as the best methods, and result in unbias statistics. The vertical line represent the μ^S.

S4.1 Simulations

We simulate 500 BETPL with a reorientation angle uniformly distributed, using the parameters $\mu^S = 1.5$, $\lambda_2^S = 0.2$ and $\lambda_1^S = 0.02$ and than we fit the synthetic trajectories with an BETPL for $\lim_a = (0.01, 0.02, 0.05, 0.1, 0.2, 0.3, 0.4, 0.5, 1, 2, 5)$. All travels are simulated with a total trip length of 5000. Analyzing the results we found that using $\lim_a$ lower than λ_2^S brings to a fitted λ_2 lower than λ_2^S and a fitted μ lower than μ^S. Instead using $\lim_a$ bigger than λ_2^S brings to a fitted λ_2 bigger than λ_2^S but a better fit of μ. This is due to the manipulation caused by the projecting algorithm of the original distribution, that tends to enhance small scales in the distribution. We want to stress that every algorithm for extracting step lengths between turning points has his own problems, eg: algorithm based on threshold in change of direction also distort the original distribution, increasing the mean step length, and can be more sensitive to noise than the projecting method. In every method the tails of the distribution are usually more distorted in particular the smaller scales can be highly sensitive also for a the finiteness of time sampling.

S4.2 Analysis with estimated μ (var_mu)

In figure S4 we plot the fitted μ for each of the gof methods. As it is possible to see the KS_tail method is the best in identifying the correct μ and is the only one unbiased ($<\mu>= 1.5 \pm 0.2$).

In figure S5 we plot the fitted λ_1 for each of the gof methods. As it is possible to see the KS_tail method is the best in identifying the correct λ_1, with a low

positive bias ($<\lambda_1>= 0.26 \pm 0.01$).

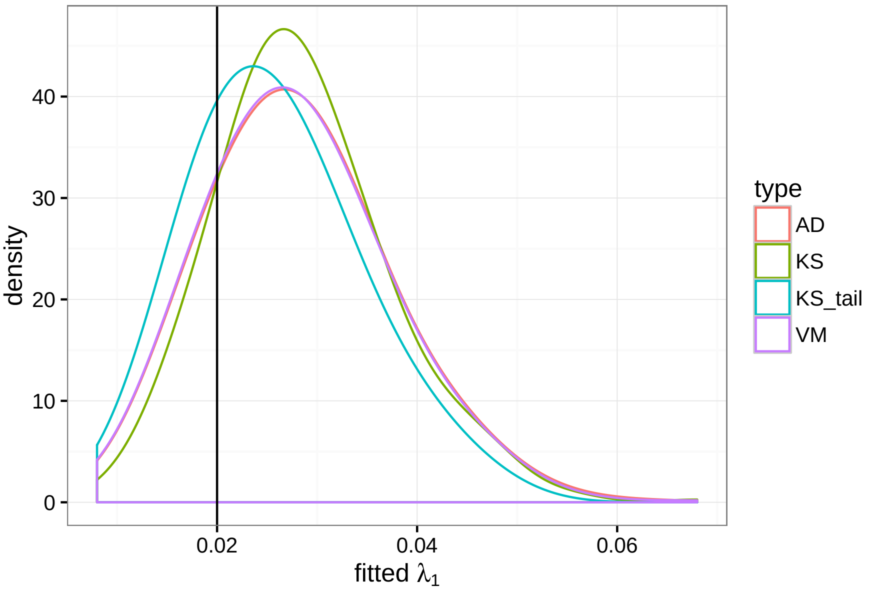

Figure S5: distribution of fitted λ_1 obtained with the different gof methods. KS_tail result as the best methods. The vertical line represent the λ_1^S.

In figure S6 we plot the fitted λ_2 for each of the gof methods. As is possible to see the KS_tail method is the worst in identifying the correct λ_2 ($<\lambda_2>= 0.9 \pm 0.7$) and the other methods especially the normal KS has better estimation of λ_2 ($<\lambda_2>= 0.6 \pm 0.6$).

Another important parameter to be considered in the choice of the best gof method is the quantity of false $\lambda_2 = 0$ fitted in the model (note the value of the intercept of the pdf with the y-axis). This is because we want to perform a log-log fit of λ_2 against the mean wind and since $\log(0) = -\infty$, value of $\lambda_2 = 0$ distort heavily the fit. As it can be seen in figure S6 all the methods have a not negligible quantity of false $\lambda_2 = 0$ fitted.

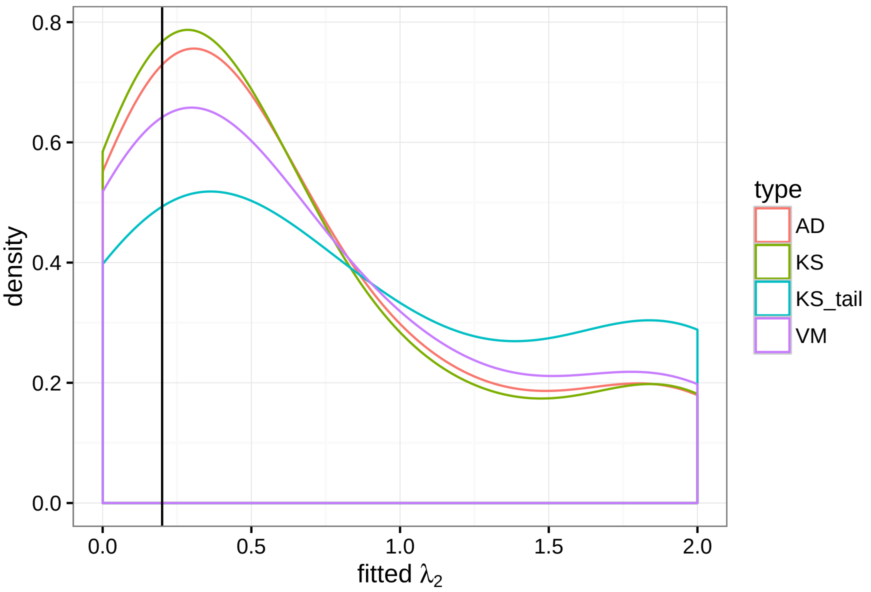

Figure S6: distribution of fitted λ_2 obtained with the different gof methods. KS results as the best fitting method. The vertical line represent the λ_2^S.

This 0-values strongly effects the log-log fit.

The three measures μ, λ_1 and λ_2 are highly correlated as can be seen in table S1 where we present the correlation coefficient for the values predicted using KS_tail as gof. It is evident that the measure of μ highly condition the measure of the other two parameters. High correlation are present for all gof methods or using just a fixed value lim_a (result not presented).

	λ_1	λ_2	μ
λ_1	1.000	-0.530	-0.830
λ_2	-0.530	1.000	0.790
μ	-0.830	0.790	1.000

Table S1: Correlation matrix of the fitted coefficients with KS_tail method.

S4.3 Analysis with imposed $\mu = 1.5$ (fix_mu)

Since the high correlation present between μ and the other two parameters we fit the synthetic trajectories fixing μ to the expected value of 1.5, we call this fit set fix_mu, compared to the normal fit set var_mu.

In figure S7 we plot the fitted λ_1 for each of the gof methods. As it is possible to see the KS_tail method is marginally the best in identifying the correct λ_1 and we obtain a better estimate of λ_1 using fix_mu statistics than var_mu ($< \lambda_1 >= 0.024 \pm 0.005$)

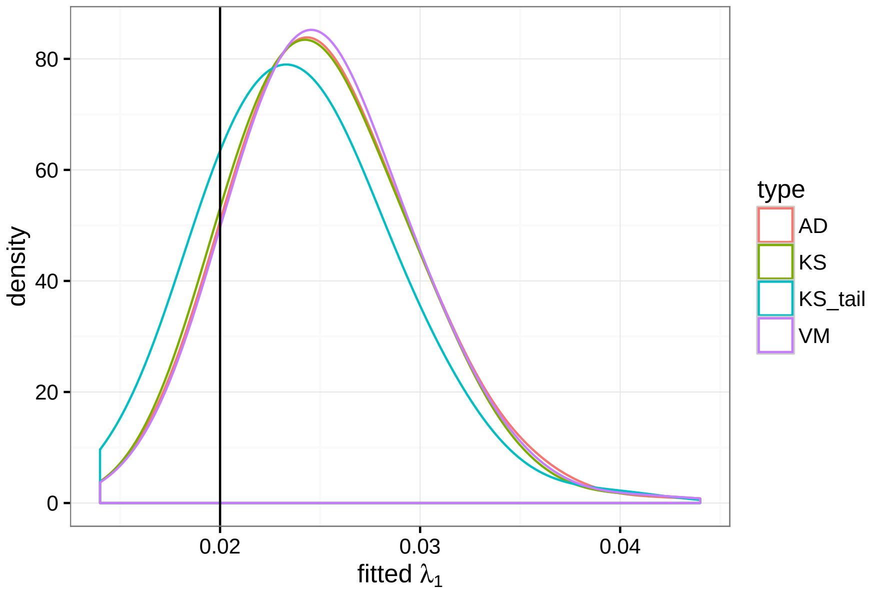

Figure S7: distribution of fitted λ_1 obtained with the different gof methods using fix_mu. KS_tail result as the best methods. The vertical line represent the λ_1^S.

In figure S8 we plot the fitted λ_2 for each of the gof methods using fix_mu. In contrast with the var_mu set the KS_tail method is the best in identifying the correct λ_2, but still with a great bias ($< \lambda_2 >= 0.85 \pm 0.5$).

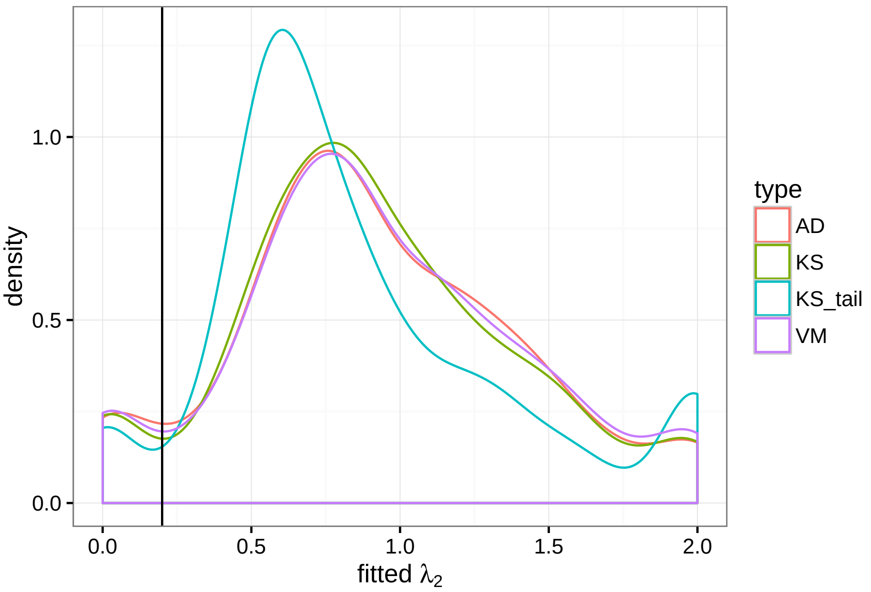

Figure S8: distribution of fitted λ_2 obtained with the different gof methods using fix_mu. KS_tail results as the best fitting method. The vertical line represent the λ_2^S.

The correlation between the two parameters remain still high (> 0.45 in KS_tail method).

Comparing the results for λ_2 in the case of fix_mu and var_mu we can note that although a more biased estimation of λ_2 in the fix_mu fit, the variance is smaller, and we avoids measure with $\lambda_2 = 0$. So it's logical to pretend that a fit with fix_mu in the real data will give a better estimate of the relationship between λ_2 and the mean wind.

S4.4 Other methods

As mentioned in the main, likelihood distribution for the parameters λ_1, λ_2 and μ is often highly skewed and as reported by Barlow (2006) the proper estimator should be the mean likelihood instead of the maximum likelihood. In order to take account of this we used as error bar of the single measures the extreme values at 95% confidence level and use the method illustrated in Barlow (2003), Barlow (2006) to calculate the mean (using just one measure) and standard deviation. Using the mean has the advantage of a lower standard deviation than using just the MLLH for λ_2 and μ and to avoids the nonphysical extreme results.

We also try to use a weighted sum of all the measure with the different $\lim_a$ with a weight, k, defined as:

$$k = \frac{1}{\sigma \mathrm{D}^{\rho}}, \tag{3}$$

where D is the gof statistics (e.g. the KS distance in case of using the KS tests), σ is the standard deviation of every measure calculated as in Barlow (2003) and $\rho \geq 0$ a parameter that interpolate between the case $\rho = 0$ of taking

all the measurement as equally important and weighted only with their σ and in the $\rho = \infty$ of taking only the measurement with best D. The idea is that for low ρ not all the measurement are to be discarded and we can take some information from all and to check consistency between the two extreme cases. For small ρ the predicted value is biased and highly depend on the choices of $\lim_a$ we use, but as always, since we are interested not in the real value of λ_2, but in how λ_2 scales with the wind, this effect can be not so important. Indeed the predicted values have the property of a small standard deviation. In figure S9 and S10 we show the evaluated λ_1 and λ_2 using $\rho = 0, 15$ and the mean likelihood and the max likelihood for the best gof. As can be seen especially for λ_2 there is not a great difference between the mean likelihood and the max likelihood, except a lower probability on the extreme of the distribution.

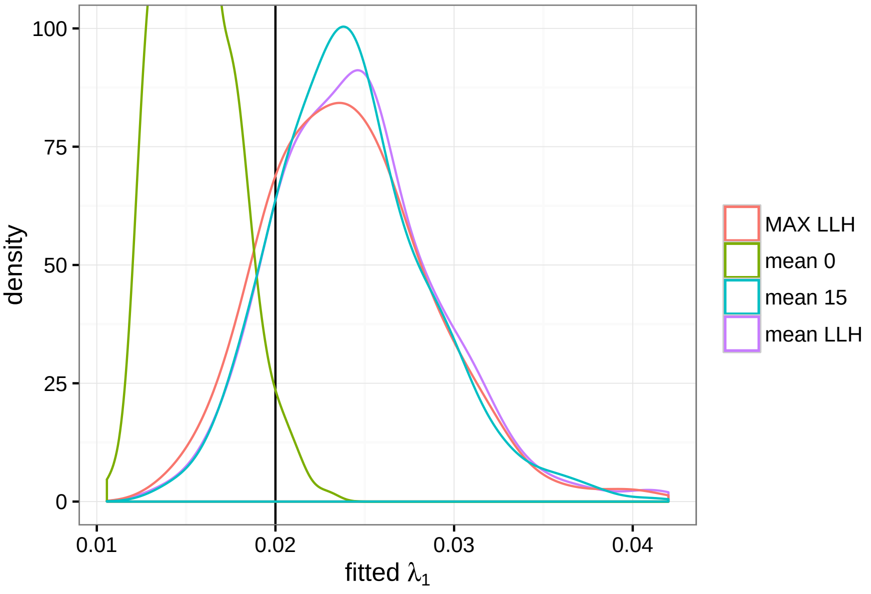

Figure S9: Distribution of fitted λ_1 with the best likelihood, the mean likelihood, the mean with $\rho = 0$ and the mean with $\rho = 15$.

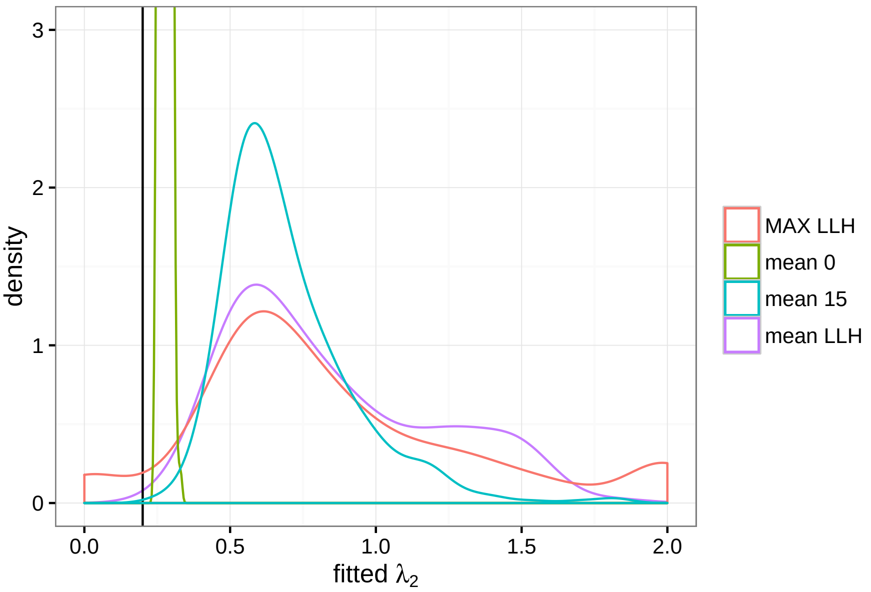

Figure S10: Distribution of fitted λ_2 with the best likelihood, the mean likelihood, the mean with $\rho = 0$ and the mean with $\rho = 15$.

Also in the discrimination between generated ETPL and BI-EXP the KS, AD and VM turns out to have worst performance respect to KS_tail.

In conclusion from simulations we founded that using the mean of the Likelihood calculated as Barlow (2006) of the single measure with best gof test (KS_tail) represent a good choice for calculation of λ_1 and λ_2.

S5 Power-laws with exponential truncation are preserved under projection

Humphries *et al.* (2013) showed that the projection of a Lévy walk is itself a Lévy walk and that projection does not result in non-Lévy walks being misidentified as Lévy walks and vice versa. This is significant advance because two- (and three-) dimension Lévy walks can now be identified in a robust, objective way if movement patterns are first projected onto the x- and y-axes to create two one-dimensional movement patterns for each individual. It is robust because turns in these projections can then be identified in an unambiguous way as occurring where the direction of travel changes. Without projection, turns can only be identified by making reference to arbitrarily defined critical-turning angles. Inappropriate choices can lead to movement patterns being wrongly classified Humphries *et al.* (2013).

Here we show that exponentially-truncated Lévy walks are also preserved under projection.

Let L be the length of a 2D step.

Then $x = L\cos(\theta) = Ls$ is the projected length where θ is the projection angle. When the projection angles are randomly and uniformed distributed

between 0 and $\pi/2$ then the distribution of θ is given by $p_\theta = 2/\pi$ and so $p_s = p_\theta \frac{d\theta}{\mathrm{ds}} = \frac{2}{\pi}\frac{1}{\sqrt{1-s^2}}$. It follows that the distribution of projected lengths

$$\begin{aligned} p_x(x) &= \int_0^1 \int_x^\infty p_L(L)\, p_s(s)\, \delta(\mathrm{Ls} - x)\, \mathrm{dLds} \\ &= \int_x^\infty p_L(L)\, p_s(x/L)\, L^{-1} \mathrm{dL} \\ &= \int_0^1 p_L(x/t)\, t^{-1} \tfrac{\mathrm{dt}}{\sqrt{1-t^2}} \end{aligned} \tag{4}$$

Some examples cases:

1. If step-lengths are exponentially distributed so that $p_L(L) = \lambda e^{-\lambda L}$ then $p_x(x) = \sqrt{\frac{2\lambda}{x\pi}} e^{-\lambda x}$ which is nearly exponential.

2. If step-lengths are power-law distributed so that $p_L(L) \propto L^{-\mu}$ then $p_x(x) \propto x^{-\mu}$, i.e., the projection of a 2D Levy walk is itself a Levy walk just has claimed by Humphries *et al.* (2013).

3. If steps-lengths are distributed according to an exponentially-truncated power-law so that
$p_L(L) \propto e^{-\lambda x} \exp L^{-\mu}$ then

$$p_x(x) \propto x^{-\mu} \int_0^1 e^{-\lambda x/t} \frac{t^{\mu-1}}{\sqrt{1-t^2}} \mathrm{dt} \tag{5}$$

This is analytically tractable when $\mu = 1$ and 2 and in these cases
$p_x(x) \approx x^{-\mu}\left(\frac{1}{6}e^{-\lambda x} + \frac{1}{2}e^{-4/3\lambda x}\right)$, i.e., the projection preserves the power-law (Lévy) exponent and to good approximation preserves the exponential-truncation.

For other values of μ, Eqn. 5 can be evaluated numerically. Two examples are shown in Fig S11.

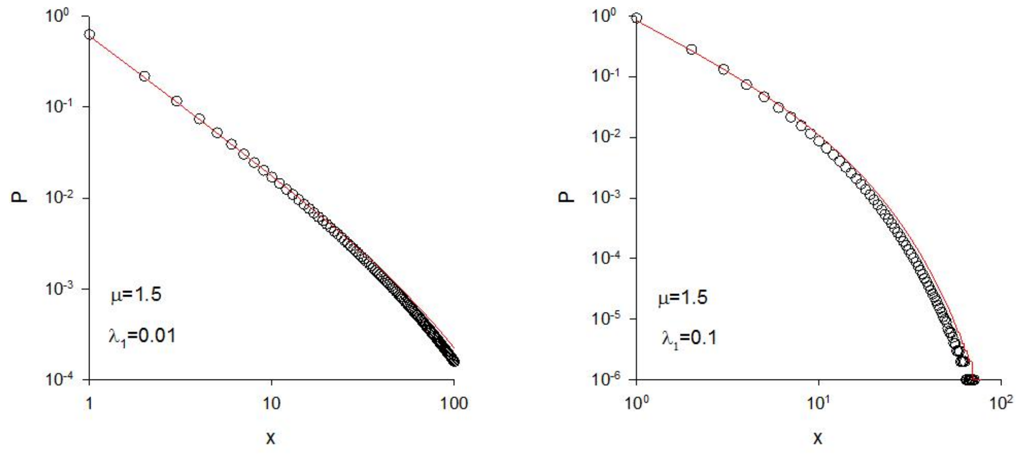

Figure S11: Comparison of $p_L(L)$ (red line) and $p_x(x)$ (o).

4. If steps-lengths are distributed according to a doubly exponentially-truncated power-law so that
$p_L\left(L\right) \propto e^{-\lambda_1 x} e^{-\lambda_2/x} L^{-\mu}$ then

$$p_x\left(x\right) \propto x^{-\mu} \int_0^1 e^{-\lambda_1 x/t} e^{-\lambda_2 t/x} \frac{t^{\mu-1}}{\sqrt{1-t^2}} \mathrm{dt}$$

This can be evaluated numerically. Two examples are shown in figure S12.

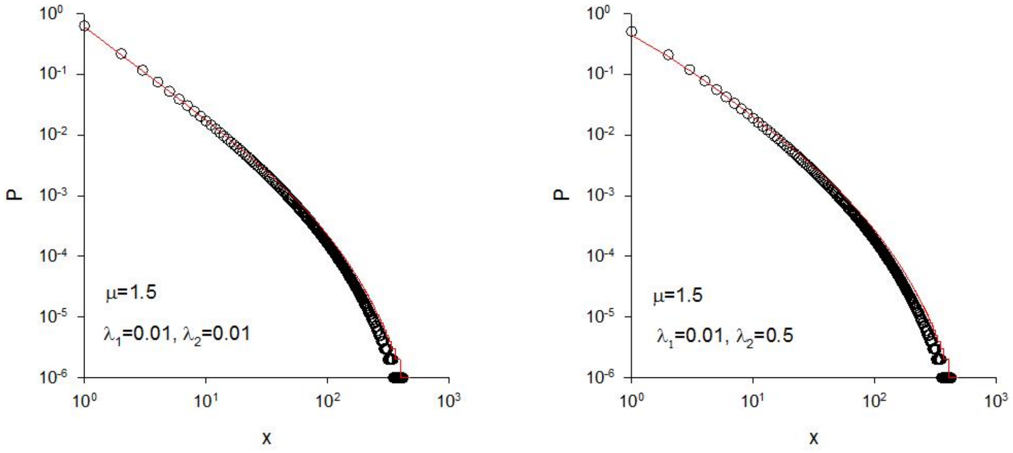

Figure S12: Comparison of $p_L(L)$ (red line) and $p_x(x)$ (o).

More pragmatically, the maximum likelihood estimates for μ and λ for projected data are close to the true values of μ and λ for the non-projected data, as illustrated in Fig. S13. The circles are projected step lengths and the black lines are fits to exponential truncated power-laws.

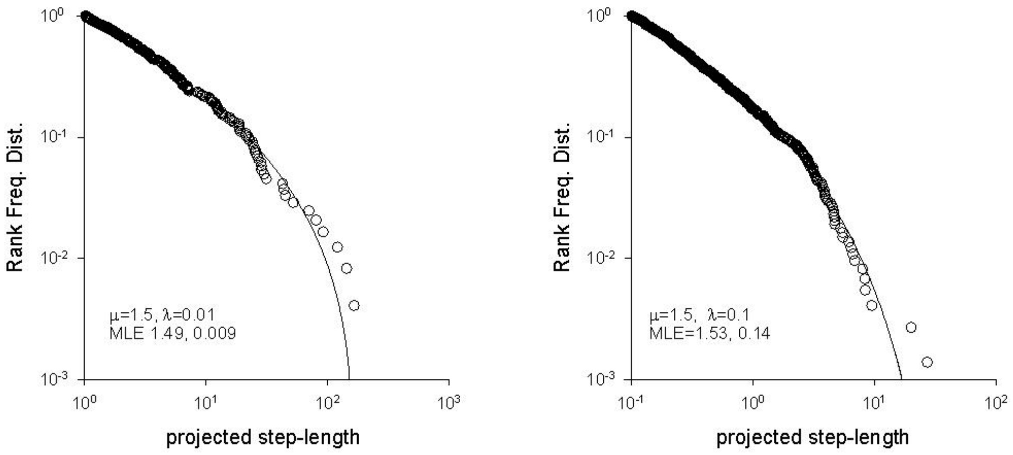

Figure S13: Comparison of true power-law exponents and exponential decay rates and maximum likelihood estimates obtained from projected data. Projected data (o) and fit (solid-lines) obtained using the maximum likelihood method.

S6 Alternative confirmation

In order to validate an other time the relationship between λ_1, λ_2 and the wind we perform a bootstrap[2] for a robust regression analysis[3] using as variables the $\log(\bar{v})$ and the fitted $\log(\lambda_1)$ and $\log(\lambda_2)$. The bootstrap also for the value of λ_1 and λ_2 predicted as a weighted sum with weights defined as in eq. 3, with ρ from 0 to 70 (as illustrated in the validation section, see S4.4). We will call β_1 and β_2 the exponents[4] in the power law between wind and λ_1 and λ_2 respectively.

S6.1 Large scale truncation

In figure S14 we present the bootstrap for ρ from 0 to 70 and the best gof, for different sub-settings in day of travels. The fit conditioned only on trips that last more than 5 days is clearly wrong and bring to false results although indicating a lower β_1 and so a stronger dependence between $\log(\lambda_1)$ and $\log(\bar{v})$ than expected. So we will exclude the lowest line in next considerations. All the other sub-settings and all the value of ρ brings to similar results and are all significative, confirming the findings reported in the main test.

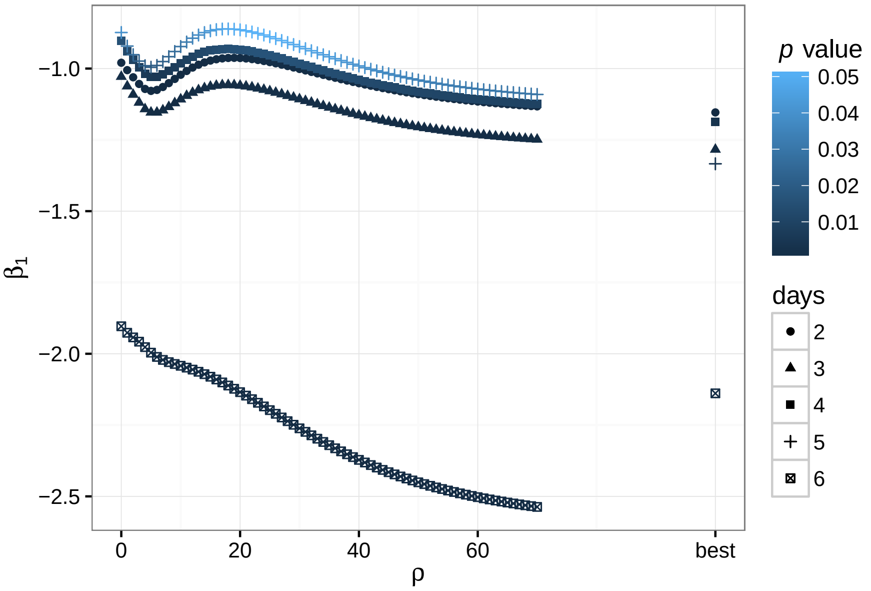

Figure S14: β_1: fitted exponent of the λ_1, $\bar{v}$ relationship for different sub-setting on trips that last more than 'days', fitting also the variable μ. ρ range from 0 to 70 and the value at "best" are the one founded using just the measure with best gof (equivalent to $\rho \to \infty$).

[2] so we sample our data-set many times randomly and perform every time the fit. Than we collect predicted parameters and calculate the mean.

[3] We used as M estimator the Huber function

[4] $\lambda_1 \sim \bar{v}^{\beta_1}$ and $\lambda_2 \sim \bar{v}^{\beta_2}$

Even in the fix_mu the line relative to days greater than 5 is clearly wrong for the same reason described above. The fits obtained using $\rho \to \infty$ estimate β_1 around -1 as theory predict, instead the fits obtained using small ρ predict a lower β_1.

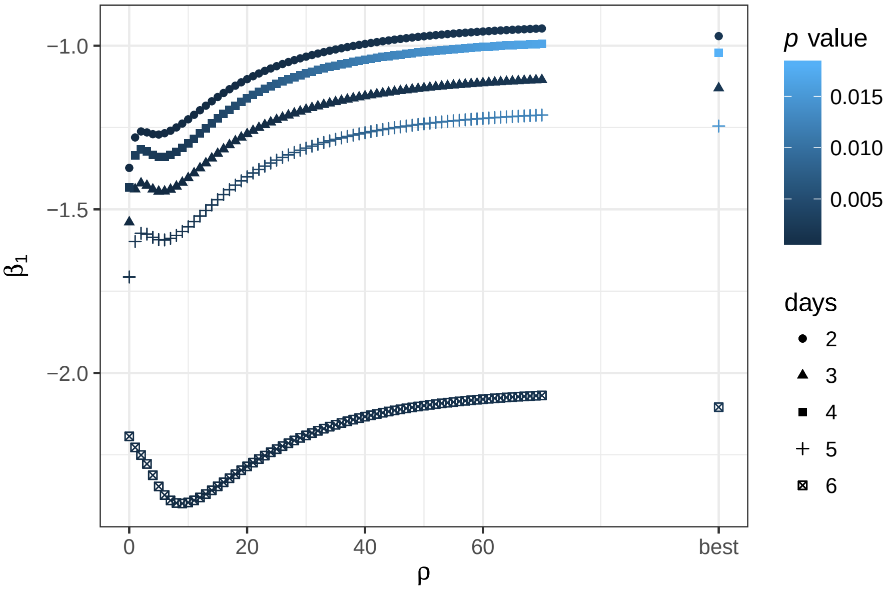

Figure S15: β_1: the fitted exponent of the λ_1, $\bar{v}$ relationship for different subsetting on trips that last more than 'days' for the fix_mu. ρ range from 0 to 70 and the value at "best" are the one founded using just the measure with best gof (equivalent to $\rho \to \infty$).

S6.2 Small scale truncation

The same operation is done for the small scale exponent λ_2. The result is showed in figure S16. Although very noisy all the bootstrap are significative and indicate a negative β_2.

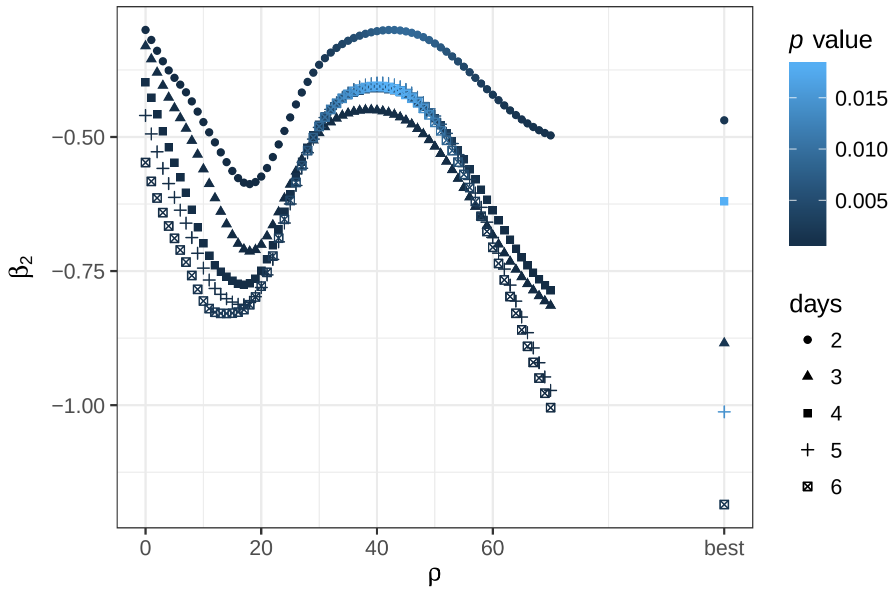

Figure S16: β_2, the fitted exponent of the λ_2 vmed relationship for different sub-setting on trips that last more than 'days' in the fix_mu case. ρ range from 0 to 70 and the value at "best" are the one founded using just the measure with best gof (equivalent to $\rho \to \infty$).

S7 Fitted values

The main statistical features of our samples are reported in Table S2 when μ is one of the parameter fitted and in Table S3 when μ is not fitted and keep at the theoretical value of 1.5.

λ_1	λ_2	μ	ETPL winning	bird identity	trip lenght	mean wind
0.063	0.500	1.375	TRUE	T72729	247.169	3.519
0.013	1.115	1.715	TRUE	T73735	559.768	3.401
0.066	0.241	1.295	TRUE	T75215	264.853	5.923
0.027	0.500	1.633	TRUE	T75237	317.101	3.518
0.022	0.831	1.407	TRUE	T75298	1629.872	3.977
0.012	0.461	1.665	TRUE	T75409	1065.063	4.661
0.066	1.055	1.157	TRUE	TH4335	363.345	4.227
0.015	0.675	1.310	TRUE	TH4336	1683.244	4.678
0.011	0.699	1.610	TRUE	TH4398	1301.538	4.768
0.122	1.038	1.232	TRUE	TH8450	340.950	2.558
0.046	0.069	1.205	TRUE	TH8510	943.373	3.384
0.037	0.500	1.385	TRUE	TH8534	1392.394	4.434
0.074	0.034	1.042	TRUE	TH8535	980.968	4.048
0.039	0.546	1.333	TRUE	TH8544	470.681	4.104
0.050	0.917	1.288	TRUE	TJ8103	375.987	2.265
0.021	1.188	1.607	TRUE	TJ8123	1341.865	2.518
0.009	1.052	1.700	TRUE	TJ8137	799.739	3.278
0.018	0.400	1.613	TRUE	TJ8150	593.335	2.679
0.018	1.643	1.782	TRUE	TJ8150	945.413	3.376
0.025	0.860	1.397	TRUE	TJ8150	811.543	3.164
0.027	0.500	1.370	TRUE	TK0421	496.604	1.579
0.014	0.285	1.528	TRUE	TK0422	1433.611	5.278
0.043	0.598	1.290	TRUE	TK0430	396.725	2.018
0.074	0.551	1.738	TRUE	TK0432	196.438	2.241
0.034	0.515	1.280	TRUE	TK0433	484.492	2.866
0.046	1.095	1.220	TRUE	TK0435	435.383	2.893
0.040	0.102	1.280	TRUE	TK0435	294.340	4.231
0.046	1.500	1.880	TRUE	TA6976	601.112	2.485
0.009	0.530	1.607	TRUE	TJ8101	1970.972	2.408
0.032	1.659	2.020	TRUE	TJ8102	695.011	7.002
0.003	1.677	1.837	TRUE	TJ8104B	1613.517	3.061
0.009	1.591	1.933	TRUE	TJ8105	918.787	2.986
0.029	0.375	1.492	TRUE	TJ8107	1834.437	3.450
0.006	1.500	1.945	TRUE	TJ8124	1410.142	4.499
0.039	0.421	1.462	TRUE	TJ8135	1252.358	2.639
0.086	0.379	1.153	TRUE	TK0417	644.195	2.423
0.043	0.174	1.495	TRUE	T97749	706.263	2.961
0.175	0.081	1.028	TRUE	T97769	416.021	4.174
0.061	0.535	1.238	TRUE	TB0524	549.704	2.846
0.085	0.036	1.040	TRUE	TC7503	813.748	2.406
0.039	0.391	1.280	TRUE	TC7506	1009.746	2.060
0.065	0.782	1.705	TRUE	TC7507	477.198	3.074
0.048	0.166	1.210	TRUE	TC7509	604.982	4.946

0.063	1.305	1.692	TRUE	TC7510	301.323	3.180
0.053	0.790	1.655	TRUE	TC7511	524.012	3.039
0.053	0.486	1.385	TRUE	TC7514	304.159	3.787
0.053	0.126	1.173	TRUE	TC7519	2212.270	3.505
0.030	0.406	1.285	TRUE	TC7521	1174.476	4.139
0.030	0.366	1.415	TRUE	TC7524	1427.996	3.657
0.030	0.328	1.220	TRUE	TC7525	944.091	3.361
0.029	0.124	1.198	TRUE	TC7526	1429.429	3.602
0.034	1.500	1.755	TRUE	TC7527	1763.283	2.144
0.024	1.597	2.130	TRUE	TC7529	510.028	4.217
0.081	1.514	1.655	TRUE	TC7533	222.466	2.754
0.111	0.823	1.258	TRUE	TE1341	957.815	1.845
0.041	1.462	1.712	TRUE	TE1342	490.569	1.319
0.009	1.300	1.540	TRUE	TE1343	2425.202	3.547
0.041	0.787	1.627	TRUE	TE1345	888.200	1.675
0.034	1.010	1.758	TRUE	TE1347	766.307	1.705
0.055	0.168	1.140	TRUE	22372	397.091	6.003
0.021	0.022	1.070	TRUE	22372	416.551	10.877
0.005	0.217	1.540	TRUE	22372	2244.327	7.115
0.058	0.500	1.837	TRUE	22388	194.425	9.228
0.126	0.560	1.360	TRUE	22388	227.108	5.936
0.145	0.642	1.370	TRUE	22388	152.087	3.995
0.076	0.500	2.130	TRUE	22388	92.382	3.086
0.158	0.041	1.228	TRUE	22388	128.572	5.107
0.064	0.059	1.175	TRUE	22525	195.114	8.097
0.089	1.285	2.002	TRUE	22525	339.744	7.888
0.080	0.263	1.325	TRUE	22525	151.151	4.788
0.019	0.287	1.458	TRUE	39109	252.478	7.463
0.033	0.490	1.482	TRUE	39109	1139.850	6.439
0.103	0.510	1.458	TRUE	39109	230.522	5.158
0.041	0.071	1.315	TRUE	39109	375.871	4.379
0.011	0.940	2.042	FALSE	39114	505.532	8.161
0.035	0.410	1.343	TRUE	39114	532.316	4.072
0.061	1.500	1.337	TRUE	44531	118.188	4.299
0.018	0.318	1.827	TRUE	44531	146.246	6.654
0.024	0.380	1.495	TRUE	44531	670.886	8.757
0.013	0.500	1.675	TRUE	44531	669.711	5.874
0.040	0.334	1.200	TRUE	44531	1357.159	4.832
0.017	0.713	1.482	TRUE	44824	319.895	4.300
0.070	0.500	1.495	TRUE	44824	225.628	9.984
0.001	0.785	1.615	TRUE	44824	2973.671	6.487
0.005	0.138	1.490	TRUE	54006	3395.396	6.323
0.013	0.079	1.423	TRUE	59360	761.243	9.332
0.084	0.782	1.292	TRUE	59360	242.862	6.747
0.093	0.015	1.048	TRUE	59360	112.924	4.522
0.082	0.451	1.255	TRUE	59360	317.408	2.663
0.071	1.232	1.883	TRUE	59755	140.621	7.323
0.001	1.052	1.528	TRUE	59755	3668.929	6.644
0.032	0.285	1.312	TRUE	61358	1053.105	6.971
0.022	0.500	1.270	TRUE	61358	369.093	6.229

0.019	0.062	1.262	TRUE	61358	603.493	4.563
0.021	0.955	1.455	TRUE	61358	278.221	4.568
0.005	0.677	1.710	TRUE	64211	3814.533	5.981
0.029	0.848	1.438	TRUE	64222	320.147	8.567
0.074	0.500	1.815	TRUE	64222	148.002	4.819
0.054	0.500	2.190	TRUE	64222	140.979	4.407
0.169	0.081	1.097	TRUE	64222	192.375	2.435
0.111	0.550	1.458	TRUE	64222	284.184	4.490
0.070	0.500	1.905	TRUE	64228	116.393	4.972
0.063	0.391	1.407	TRUE	64228	461.091	7.400
0.023	0.662	1.968	TRUE	64228	500.800	5.945
0.035	0.056	1.125	TRUE	64228	752.345	3.243
0.003	1.347	1.405	TRUE	64338	2942.069	6.289
0.017	1.222	1.677	TRUE	64391	601.095	5.735
0.006	0.075	1.320	TRUE	64391	3905.333	6.442
0.097	0.500	1.532	TRUE	64393	373.872	6.414
0.068	1.040	1.337	TRUE	64393	318.380	8.490
0.033	0.555	1.545	TRUE	64393	554.733	8.008
0.029	0.905	1.740	TRUE	64393	482.544	8.228
0.060	0.124	1.225	TRUE	64393	744.270	3.872
0.141	1.500	1.577	TRUE	64397	88.655	4.350
0.062	0.405	1.440	TRUE	64397	591.570	8.772
0.026	0.323	1.478	TRUE	64397	1493.510	6.055
0.009	0.474	1.650	TRUE	67769	451.468	5.938
0.001	0.557	1.603	TRUE	67769	4383.201	6.936
0.054	0.181	1.360	TRUE	73254	427.339	8.230
0.150	1.500	1.610	TRUE	73254	129.861	7.148
0.026	1.500	2.035	TRUE	73254	387.173	7.160
0.068	1.268	1.590	TRUE	73254	290.180	3.475
0.053	1.198	1.220	TRUE	73254	566.605	4.663
0.019	1.485	1.633	TRUE	73290	828.739	7.116
0.017	0.217	1.625	TRUE	73290	1242.688	6.874
0.029	1.030	1.613	TRUE	73290	929.651	3.582
0.147	1.105	1.440	TRUE	76503	85.031	4.703
0.022	0.271	1.228	FALSE	76503	284.045	6.439
0.031	0.635	1.155	TRUE	76503	403.702	7.631
0.046	0.159	1.157	FALSE	76503	2040.941	6.041
0.064	1.280	1.242	TRUE	76503	448.104	3.997
0.100	0.500	1.403	FALSE	76516	571.762	8.533
0.029	0.200	1.390	TRUE	76516	209.089	5.069
0.013	0.500	1.663	TRUE	76516	376.297	4.569
0.017	0.154	1.272	FALSE	76519	1762.391	5.856
0.035	0.590	1.170	TRUE	76519	1170.592	5.219
0.022	0.682	1.942	FALSE	76603	403.865	7.876
0.045	0.013	1.240	FALSE	76603	224.220	6.955
0.035	0.780	1.163	TRUE	76603	890.380	5.185
0.043	0.072	1.235	TRUE	76603	707.824	3.536
0.124	0.833	1.545	FALSE	78615	107.463	4.930
0.020	0.196	1.657	TRUE	78615	200.403	7.582
0.058	0.087	1.240	TRUE	78615	893.293	6.031

0.021	0.226	1.262	TRUE	78615	486.015	7.129
0.067	0.500	1.425	TRUE	78615	436.182	3.489
0.077	0.500	1.327	TRUE	78615	286.502	4.772
0.022	0.400	1.500	TRUE	78646	1696.891	6.122
0.012	0.620	1.607	FALSE	8043	1067.080	9.453
0.087	0.050	1.077	TRUE	8043	410.918	5.355
0.086	0.323	1.627	FALSE	8043	127.340	4.739
0.021	0.610	1.470	FALSE	L045564	953.586	7.248
0.020	0.193	1.420	TRUE	L045564	268.796	8.103
0.007	0.980	1.452	TRUE	L045564	532.692	9.382
0.026	0.147	1.423	FALSE	L045564	363.161	7.506
0.011	0.253	1.575	TRUE	L045564	302.126	7.213
0.005	0.500	1.552	TRUE	L045564	2243.342	9.921
0.035	0.233	1.653	TRUE	L053613	343.623	5.207
0.023	0.696	1.742	TRUE	L053613	413.150	3.991
0.018	0.333	1.380	TRUE	L053613	768.076	5.200
0.076	0.177	1.255	TRUE	L059554	300.651	5.232
0.015	1.150	1.183	TRUE	L059554	420.074	4.807
0.040	0.500	1.720	TRUE	L059554	164.843	2.896
0.047	0.713	1.323	TRUE	L059554	317.859	4.590
0.018	0.237	1.228	TRUE	L059554	703.772	3.409
0.152	0.940	1.730	TRUE	L44221	114.197	5.259
0.037	0.500	1.667	TRUE	L44221	115.080	4.981
0.134	0.041	1.115	TRUE	L44221	87.513	4.980
0.026	0.802	1.650	TRUE	L44221	159.035	6.085
0.029	0.500	1.285	TRUE	L44221	230.289	2.245
0.006	0.706	1.623	TRUE	L44255	243.619	2.462
0.005	0.246	1.545	TRUE	L44255	2346.600	5.302
0.015	1.167	1.363	TRUE	L44255	285.647	7.315
0.004	0.703	1.345	TRUE	CRlog100F	2429.803	4.418
0.046	0.875	1.500	FALSE	CRlog110F	676.334	5.247
0.010	0.953	1.468	TRUE	CRlog110F	2548.454	5.157
0.009	0.557	1.468	FALSE	CRlog118F	783.464	2.811
0.004	1.500	1.903	TRUE	CRlog118F	942.558	10.346
0.006	0.500	1.567	FALSE	CRlog118F	1742.797	4.924
0.004	0.902	1.597	TRUE	CRlog119F	5231.414	5.069
0.011	0.501	1.100	TRUE	CRlog34F	774.119	5.828
0.001	1.008	1.718	TRUE	CRlog34F	2256.600	2.522
0.004	0.544	1.198	TRUE	CRlog88F	2459.662	6.699
0.016	0.542	1.353	TRUE	CRlog92F	1291.719	6.604
0.015	0.182	1.232	TRUE	CRlog92F	1132.604	3.842
0.021	0.101	1.133	TRUE	CRlog92F	632.367	6.475
0.030	0.114	1.315	FALSE	INlog100F	498.047	4.738
0.004	0.621	1.420	TRUE	INlog104F	2806.140	3.368
0.002	0.500	1.417	FALSE	INlog79F	1103.057	3.369
0.006	0.438	1.420	TRUE	20101	1002.089	7.270
0.017	0.176	1.360	TRUE	43992	2739.793	7.008
0.012	1.575	1.815	TRUE	74835	2369.543	7.139
0.002	0.419	1.782	TRUE	76429	1100.034	7.914
0.007	1.556	1.655	TRUE	76430	2042.334	7.541

0.006	1.500	1.817	TRUE	76434	1709.780	7.367
0.002	0.965	1.850	FALSE	76436	1237.844	8.558
0.006	0.205	1.192	FALSE	76436	574.678	6.691
0.029	0.500	1.212	FALSE	78383	271.569	8.887
0.007	0.270	1.545	FALSE	78383	962.053	7.440
0.006	0.670	1.585	TRUE	78384	1564.158	6.504
0.007	1.500	1.827	FALSE	78386	1646.809	6.886

Table S2: Fitted values of $\lambda_1, \lambda_2, \mu$, if the BETPL is winning against a biexponential. This values are fitted in case of variable μ. The other plotted values are: mean wind along the trajectory, length of the trip, and a code identificative of the bird.

λ_1	λ_2	ETPL winning	bird identity	trip lenght	mean wind
0.039	0.227	TRUE	T72729	247.169	3.519
0.013	0.434	TRUE	T73735	559.768	3.401
0.031	0.546	TRUE	T75215	264.853	5.923
0.021	0.348	TRUE	T75237	317.101	3.518
0.018	0.989	TRUE	T75298	1629.872	3.977
0.014	0.208	TRUE	T75409	1065.063	4.661
0.055	2.329	TRUE	TH4335	363.345	4.227
0.012	1.487	TRUE	TH4336	1683.244	4.678
0.011	0.324	TRUE	TH4398	1301.538	4.768
0.051	0.568	TRUE	TH8450	340.950	2.558
0.022	0.293	TRUE	TH8510	943.373	3.384
0.025	0.311	TRUE	TH8534	1392.394	4.434
0.042	0.576	FALSE	TH8535	980.968	4.048
0.015	0.316	TRUE	TH8544	470.681	4.104
0.032	1.800	TRUE	TJ8103	375.987	2.265
0.028	1.007	TRUE	TJ8123	1341.865	2.518
0.014	0.481	TRUE	TJ8137	799.739	3.278
0.025	0.272	FALSE	TJ8150	593.335	2.679
0.021	0.518	TRUE	TJ8150	945.413	3.376
0.015	1.110	TRUE	TJ8150	811.543	3.164
0.011	0.305	TRUE	TK0421	496.604	1.579
0.014	0.340	TRUE	TK0422	1433.611	5.278
0.031	1.457	TRUE	TK0430	396.725	2.018
0.125	0.750	TRUE	TK0432	196.438	2.241
0.013	1.061	TRUE	TK0433	484.492	2.866
0.034	2.298	TRUE	TK0435	435.383	2.893
0.044	1.909	TRUE	TK0435	294.340	4.231
0.070	0.881	TRUE	TA6976	601.112	2.485
0.025	0.554	TRUE	TJ8101	1970.972	2.408
0.043	1.037	TRUE	TJ8102	695.011	7.002
0.011	0.223	TRUE	TJ8104B	1613.517	3.061
0.029	0.706	TRUE	TJ8105	918.787	2.986
0.025	0.460	TRUE	TJ8107	1834.437	3.450
0.018	0.737	TRUE	TJ8124	1410.142	4.499
0.032	0.814	TRUE	TJ8135	1252.358	2.639
0.070	1.508	FALSE	TK0417	644.195	2.423
0.048	0.175	FALSE	T97749	706.263	2.961
0.071	0.199	TRUE	T97769	416.021	4.174
0.027	0.426	FALSE	TB0524	549.704	2.846
0.050	0.568	TRUE	TC7503	813.748	2.406
0.017	0.503	TRUE	TC7506	1009.746	2.060
0.062	1.062	TRUE	TC7507	477.198	3.074
0.026	0.482	TRUE	TC7509	604.982	4.946
0.045	0.318	TRUE	TC7510	301.323	3.180
0.053	0.507	TRUE	TC7511	524.012	3.039
0.043	0.844	TRUE	TC7514	304.159	3.787
0.025	0.857	TRUE	TC7519	2212.270	3.505
0.016	0.689	TRUE	TC7521	1174.476	4.139

0.026	0.430	TRUE	TC7524	1427.996	3.657
0.010	0.157	TRUE	TC7525	944.091	3.361
0.023	0.900	TRUE	TC7526	1429.429	3.602
0.034	0.958	TRUE	TC7527	1763.283	2.144
0.060	0.301	TRUE	TC7529	510.028	4.217
0.086	1.727	TRUE	TC7533	222.466	2.754
0.091	2.220	TRUE	TE1341	957.815	1.845
0.025	1.067	TRUE	TE1342	490.569	1.319
0.011	1.300	TRUE	TE1343	2425.202	3.547
0.037	0.465	TRUE	TE1345	888.200	1.675
0.055	0.614	TRUE	TE1347	766.307	1.705
0.026	0.543	TRUE	22372	397.091	6.003
0.004	0.140	TRUE	22372	416.551	10.877
0.006	0.139	TRUE	22372	2244.327	7.115
0.052	0.393	TRUE	22388	194.425	9.228
0.114	1.893	TRUE	22388	227.108	5.936
0.112	0.502	TRUE	22388	152.087	3.995
0.115	0.750	FALSE	22388	92.382	3.086
0.141	0.653	TRUE	22388	128.572	5.107
0.024	0.140	TRUE	22525	195.114	8.097
0.130	0.472	TRUE	22525	339.744	7.888
0.052	0.163	TRUE	22525	151.151	4.788
0.007	0.091	TRUE	39109	252.478	7.463
0.011	0.245	TRUE	39109	1139.850	6.439
0.090	1.307	TRUE	39109	230.522	5.158
0.063	0.423	TRUE	39109	375.871	4.379
0.024	0.417	TRUE	39114	505.532	8.161
0.021	0.324	TRUE	39114	532.316	4.072
0.041	2.250	TRUE	44531	118.188	4.299
0.026	0.262	TRUE	44531	146.246	6.654
0.022	0.379	TRUE	44531	670.886	8.757
0.017	0.064	TRUE	44531	669.711	5.874
0.024	0.936	TRUE	44531	1357.159	4.832
0.010	0.860	TRUE	44824	319.895	4.300
0.064	0.800	TRUE	44824	225.628	9.984
0.002	0.208	FALSE	44824	2973.671	6.487
0.003	0.093	TRUE	54006	3395.396	6.323
0.008	0.224	TRUE	59360	761.243	9.332
0.041	0.395	TRUE	59360	242.862	6.747
0.125	2.250	TRUE	59360	112.924	4.522
0.056	0.808	TRUE	59360	317.408	2.663
0.087	0.946	TRUE	59755	140.621	7.323
0.002	0.668	TRUE	59755	3668.929	6.644
0.009	0.223	TRUE	61358	1053.105	6.971
0.011	1.032	TRUE	61358	369.093	6.229
0.021	0.509	TRUE	61358	603.493	4.563
0.022	0.055	TRUE	61358	278.221	4.568
0.009	0.085	TRUE	64211	3814.533	5.981
0.018	0.303	TRUE	64222	320.147	8.567
0.077	0.750	TRUE	64222	148.002	4.819

0.103	0.750	TRUE	64222	140.979	4.407
0.097	0.230	TRUE	64222	192.375	2.435
0.104	1.698	TRUE	64222	284.184	4.490
0.088	0.750	TRUE	64228	116.393	4.972
0.047	0.400	TRUE	64228	461.091	7.400
0.046	0.241	TRUE	64228	500.800	5.945
0.020	0.588	TRUE	64228	752.345	3.243
0.001	0.135	TRUE	64338	2942.069	6.289
0.017	0.547	TRUE	64391	601.095	5.735
0.003	0.371	TRUE	64391	3905.333	6.442
0.060	0.750	TRUE	64393	373.872	6.414
0.053	2.250	TRUE	64393	318.380	8.490
0.030	0.395	FALSE	64393	554.733	8.008
0.032	0.439	TRUE	64393	482.544	8.228
0.049	1.234	TRUE	64393	744.270	3.872
0.135	2.250	FALSE	64397	88.655	4.350
0.049	0.349	TRUE	64397	591.570	8.772
0.028	0.108	TRUE	64397	1493.510	6.055
0.012	0.991	FALSE	67769	451.468	5.938
0.002	0.299	FALSE	67769	4383.201	6.936
0.037	0.835	TRUE	73254	427.339	8.230
0.157	2.250	TRUE	73254	129.861	7.148
0.036	0.298	TRUE	73254	387.173	7.160
0.063	1.511	TRUE	73254	290.180	3.475
0.031	0.234	TRUE	73254	566.605	4.663
0.019	1.397	TRUE	73290	828.739	7.116
0.022	0.275	TRUE	73290	1242.688	6.874
0.032	1.035	TRUE	73290	929.651	3.582
0.092	0.228	TRUE	76503	85.031	4.703
0.015	0.376	FALSE	76503	284.045	6.439
0.020	1.849	TRUE	76503	403.702	7.631
0.020	0.865	FALSE	76503	2040.941	6.041
0.044	2.250	TRUE	76503	448.104	3.997
0.066	0.718	FALSE	76516	571.762	8.533
0.019	0.239	TRUE	76516	209.089	5.069
0.019	0.821	TRUE	76516	376.297	4.569
0.010	0.616	FALSE	76519	1762.391	5.856
0.015	0.143	TRUE	76519	1170.592	5.219
0.074	2.250	FALSE	76603	403.865	7.876
0.017	0.051	FALSE	76603	224.220	6.955
0.020	1.994	TRUE	76603	890.380	5.185
0.019	0.169	TRUE	76603	707.824	3.536
0.107	0.574	FALSE	78615	107.463	4.930
0.028	0.589	TRUE	78615	200.403	7.582
0.024	0.600	TRUE	78615	893.293	6.031
0.011	0.472	TRUE	78615	486.015	7.129
0.058	1.658	TRUE	78615	436.182	3.489
0.036	0.954	TRUE	78615	286.502	4.772
0.022	0.403	TRUE	78646	1696.891	6.122
0.018	0.332	FALSE	8043	1067.080	9.453

0.038	0.293	TRUE	8043	410.918	5.355
0.128	0.702	FALSE	8043	127.340	4.739
0.016	0.357	FALSE	L045564	953.586	7.248
0.018	0.974	TRUE	L045564	268.796	8.103
0.005	0.385	TRUE	L045564	532.692	9.382
0.019	0.160	FALSE	L045564	363.161	7.506
0.016	2.250	TRUE	L045564	302.126	7.213
0.006	0.308	TRUE	L045564	2243.342	9.921
0.045	0.110	TRUE	L053613	343.623	5.207
0.033	0.216	TRUE	L053613	413.150	3.991
0.011	0.425	TRUE	L053613	768.076	5.200
0.046	0.351	TRUE	L059554	300.651	5.232
0.005	0.205	TRUE	L059554	420.074	4.807
0.031	0.497	TRUE	L059554	164.843	2.896
0.044	2.538	TRUE	L059554	317.859	4.590
0.015	1.187	TRUE	L059554	703.772	3.409
0.165	0.908	TRUE	L44221	114.197	5.259
0.030	0.387	TRUE	L44221	115.080	4.981
0.072	0.144	TRUE	L44221	87.513	4.980
0.021	1.558	TRUE	L44221	159.035	6.085
0.017	0.715	TRUE	L44221	230.289	2.245
0.013	0.406	FALSE	L44255	243.619	2.462
0.008	0.478	TRUE	L44255	2346.600	5.302
0.010	2.030	FALSE	L44255	285.647	7.315
0.002	0.240	TRUE	CRlog100F	2429.803	4.418
0.017	0.195	FALSE	CRlog110F	676.334	5.247
0.010	1.019	TRUE	CRlog110F	2548.454	5.157
0.010	0.582	FALSE	CRlog118F	783.464	2.811
0.009	0.452	TRUE	CRlog118F	942.558	10.346
0.008	0.366	FALSE	CRlog118F	1742.797	4.924
0.005	0.461	TRUE	CRlog119F	5231.414	5.069
0.003	1.632	TRUE	CRlog34F	774.119	5.828
0.003	0.450	TRUE	CRlog34F	2256.600	2.522
0.001	1.440	TRUE	CRlog88F	2459.662	6.699
0.012	0.716	TRUE	CRlog92F	1291.719	6.604
0.009	0.563	TRUE	CRlog92F	1132.604	3.842
0.013	1.409	TRUE	CRlog92F	632.367	6.475
0.026	0.255	FALSE	INlog100F	498.047	4.738
0.005	0.875	TRUE	INlog104F	2806.140	3.368
0.005	0.591	FALSE	INlog79F	1103.057	3.369
0.004	0.470	TRUE	20101	1002.089	7.270
0.008	0.277	FALSE	43992	2739.793	7.008
0.011	0.714	TRUE	74835	2369.543	7.139
0.005	0.068	TRUE	76429	1100.034	7.914
0.008	1.222	TRUE	76430	2042.334	7.541
0.016	1.112	FALSE	76434	1709.780	7.367
0.008	0.163	FALSE	76436	1237.844	8.558
0.002	0.137	FALSE	76436	574.678	6.691
0.018	2.250	FALSE	78383	271.569	8.887
0.009	0.275	FALSE	78383	962.053	7.440

0.007	0.316	FALSE	78384	1564.158	6.504
0.008	0.717	FALSE	78386	1646.809	6.886

Table S3: Fitted values of λ_1, λ_2 and if the BETPL is winning against a biexponential. This values are fitted in case of $\mu = 1.5$. The other values are: mean wind along the trajectory, length of the trip, and a code identificative of the bird.

S8 Relationship μ with the wind

The variable μ has no correlation with the wind as can be seen in figure S17.

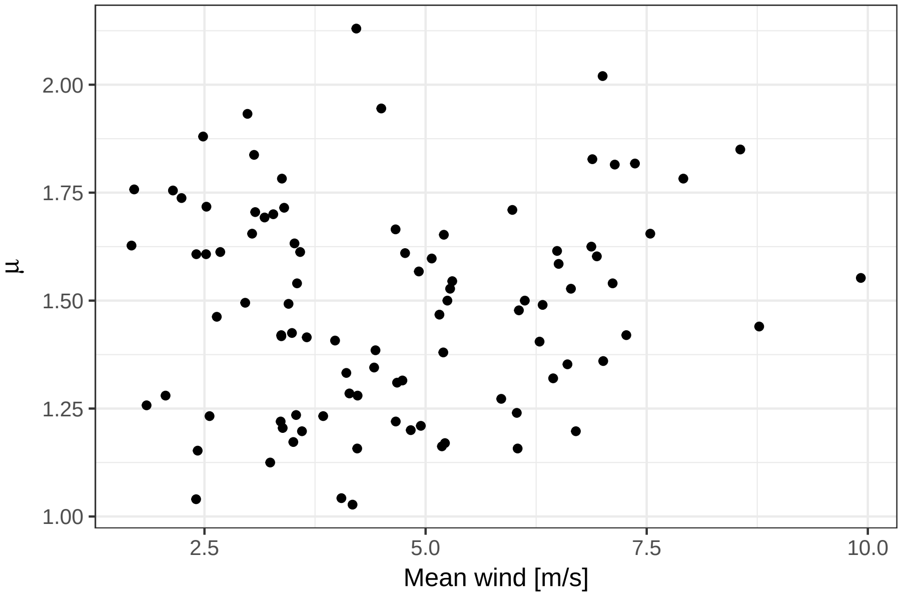

Figure S17: Plot of μ against the mean wind for trips that last more than two days.

In figure S18 we plot the mean squared error from the predicted value 1.5 dividing the data-set using the Maximum distance from the colony reached in the travel. As is possible to see the fitting converge to 1.5, with the increase of maximum displacement. The extreme of the intervals used for the subsetting are (0,250,500,750,1000, 1250+ [Km]).

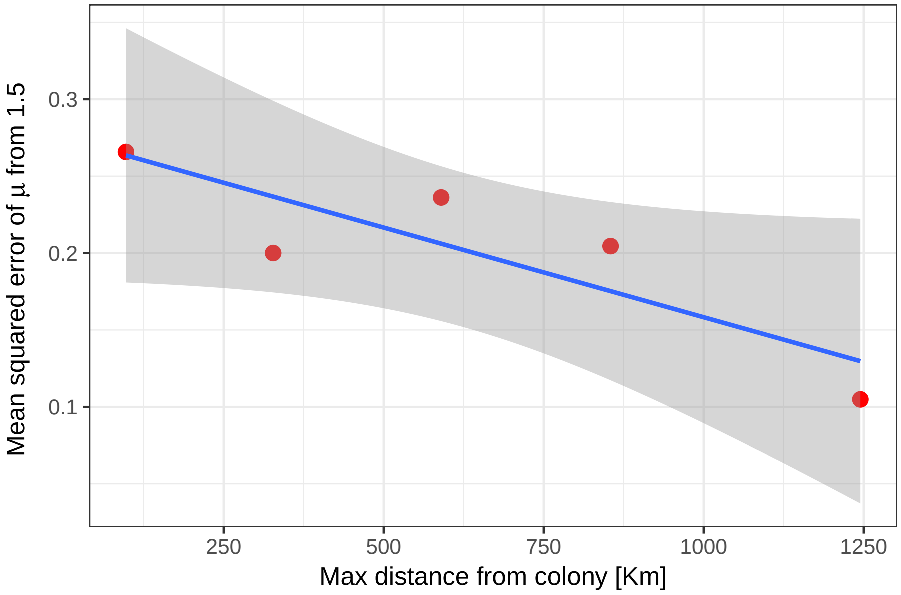

Figure S18: Mean square error against the predicted value 1.5. A linear fit of all the points is plotted. Can be easily see that the error decay almost linearly with the maximum displacement.

S9 Mechanistic wind effects on trajectories

In principle the observed correlations between λ_1 and λ_2 and the wind speed could be originated mechanistically by the impact of wind on the movement of birds Paiva *et al.* (2010). In particular we could expect that head-wind could reduce and tail wind could impact sistematically both the cut off values (namelly increasing λ_2 and decreasing λ_1). We have no specific predictions for cross winds. To test for this effect, for each bird we computed the average projection of the wind on the movement direction ($< v^{\parallel} >$) and evaluated how much this variable could explain the value of the cut offs. If the value of the cut offs is only determined by the mechanistic effect of wind we should find a significative effect and a model with both wind speed and $< v^{\parallel} >$ should outperform the models reported in figure 3 and 4 of the main text. Inversely, a non significant $< v^{\parallel} >$ correlation mean that the mechanistic effect of wind direction is not very relevant.

For all possible days of travel subsets we have two cases: or the best aic is found using the model with only the mean wind, selecting so our model, or the best aic is found using $\log(< v >)$ and $< v^{\parallel} >$, but still the significance and the standardized coefficient of the term $< v^{\parallel} >$ is poor compared to the one of $< v >$, meaning that a mechanicistic effect of the wind if present, is much less relevant than the olfactory navigation in determining the values of both λ_1 and λ_2. Regarding λ_1, subsetting the dataset for travels that last more than four days (subset where there is the greatest advantage for the alternative model

among all the subsets) we have a Δ AIC of -1.3 for the model $\lambda_1 \sim \log(<v>) + <v^{\parallel}>$, but the ratio of the coefficients of the glm between $\log(<v>)$ and $<v^{\parallel}>$ is 2.5 and the p.values of $\log(<v>)$ and $<v^{\parallel}>$ is respectively: $p < 0.0001$ and $p = 0.018$.

Regarding λ_2 sub-setting the data-set for travels that last more than four days, the Δ AIC is 1.99 and the model should be rejected.

S10 Model validation using data from indipendent data

To challenge our method of data analysis we decided to use the data provided by Pollonara *et al.* (2015). These authors investigated the homing performance of Scopoli's shearwaters (*Calonectris diomedea*) breeding at Pianosa island (Italy). These data are of public domain and were downloaded from MoveBank (https://www.movebank.org) accessed on the 30/7/2017. The following data were available: 11 anosmic, 8 control and 9 magnetically-disrupted birds. The experimental methods used are fully reported in the original paper. After treatment, birds were released in the Lion Gulf, 400 km west of the colony. The data set was reduced to meet the statistical exigencies of our analysis because several trajectories were not complete and so were discarded. Thus our sample is constituted by 6 anosmic bird trajectories, 8 control bird trajectories and 7 magnetically-disrupted bird trajectories. Since in many cases the available statistics was not enough to perform our analysis on an individual basis (the trajectory are quite short in time compare to ours), we decided to pool the trajectories with identical treatment. The pooling produced a statistics of around 1500 step lengths for the anosmic birds, 750 step lengths for the control group birds, and 770 step lengths for the magnetically disrupted birds. We fit the data with the double exponentially truncated Lévy model and the goodness-of-fit was tested using the Kolmogorov-Smirnov test weighted for tails, KS_{tail} (cf. supplementary matherials section S4 for details). Results are displayed in Figure S19.

The KS_{tail} gof values get stable for $lim_a > 0.25$ km. It is clear that control birds outperform the test with respect to anosmic birds and at some extent with respect to magnetically disrupted birds. We wish to stress that such results are derived using exactly the same software which was employed for the analyses in our paper. Using an independent data set, this result confirms that our modelling approach is able to identify the presence of olfactory navigation under natural conditions discriminating trajectories of birds using olfactory navigation from birds using different navigation mechanisms. Both our analysis and the one by Pollonara *et al.* (2015) concur to the same conclusion that anosmic birds present an impaired navigational mechanism. However in our analysis magnetic-deprived birds appear closer to anosmic birds while Pollonara *et al.* (2015) stressed that even magnetic-deprived birds navigate better than anosmic ones. However we draw attention to the plots relative to the initial orientation of birds (Fig. 3 in Pollonara *et al.* (2015)) showing the initial homeward orientation. Control birds were well orientated towards home direction, while anosmic were significantly oriented elsewhere and magnetic-deprived birds where only marginally oriented towards home. The bivariate distribution of magnetic-

deprived birds appeared to be more scattered than the one of control birds. This pattern fits very well with what we observed. We conclude that both our modelling approach and the experimental approach of Pollonara et all 2015. Get to identical conclusion.

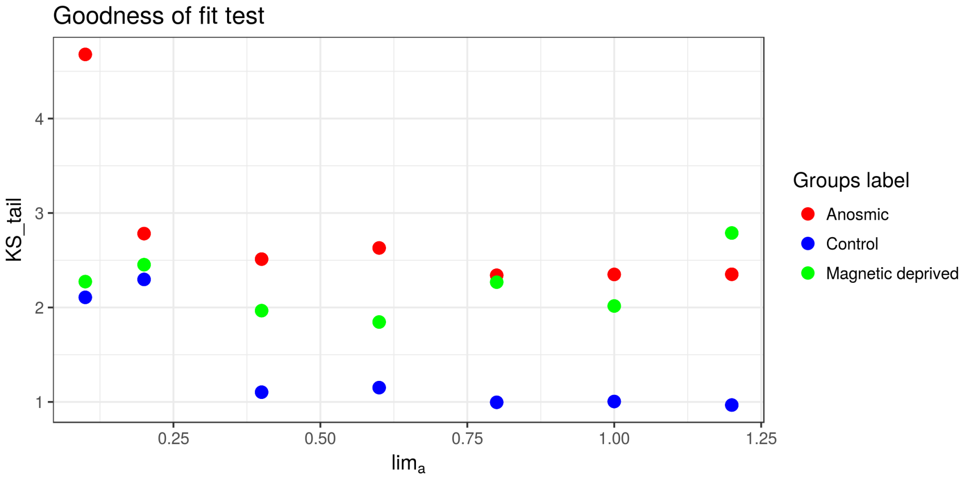

Figure S19: We display the Kolmogorov-Smirnoff statistics corrected for tails, KS_{tail}, (smaller is better) (ordinates) as a function of lim_a. The value of lim_a (km) is the value under which we cut the distribution, since there is an intrinsic noise at small scales in GPS tracks and in the identification of step lengths (validation in supplementary materials section S4).

References

Barlow, R. (2003). Asymmetric systematic errors. *arXiv preprint physics 0306138*.

Barlow, R. (2006). Asymmetric statistical errors. In: *Statistical Problems in Particle Physics, Astrophysics and Cosmology*. Imperial college press, pp. 56–59.

Humphries, N.E., Weimerskirch, H. & Sims, D.W. (2013). A new approach for objective identification of turns and steps in organism movement data relevant to random walk modelling. *Methods Ecol. Evol.*, 4, 930–938.

Nyberg, M., Ambjörnsson, T. & Lizana, L. (2016). A simple method to calculate first-passage time densities with arbitrary initial conditions. *New Journal of Physics*, 18, 063019.

Paiva, V.H., Guilford, T., Meade, J., Geraldes, P., Ramos, J.A. & Garthe, S. (2010). Flight dynamics of cory's shearwater foraging in a coastal environment. *Zoology*, 113, 47–56.

Pollonara, E., Luschi, P., Guilford, T., Wikelski, M., Bonadonna, F. & Gagliardo, A. (2015). Olfaction and topography, but not magnetic cues, control navigation in a pelagic seabird: displacements with shearwaters in the Mediterranean Sea. *Sci. Rep.*, 5.

Reynolds, A.M., Cecere, J.G., Paiva, V.H., Ramos, J.A. & Focardi, S. (2015). Pelagic seabird flight patterns are consistent with a reliance on olfactory maps for oceanic navigation. *Proc. R. Soc. B*, 282.
